# Accelerometer-derived real-world sleep stages and risk of incident diseases: A UK Biobank cohort study and phenome-wide association analysis

**DOI:** 10.1371/journal.pmed.1005213

**Published:** 2026-09-17

**Authors:** Jingsong Luo, Ruiyi Liu, Jie Yin, Wangnan Cao, Shengzhi Sun, Rui Chen

**Affiliations:** 1 School of Public Health, Peking University, Beijing, China; 2 School of Public Health, Capital Medical University, Beijing, China; 3 Beijing Key Laboratory of Environment and Aging, Capital Medical University, Beijing, China; 4 Beijing Laboratory of Allergic Diseases, Beijing Municipal Education Commission, Beijing, China; UNITED KINGDOM OF GREAT BRITAIN AND NORTHERN IRELAND

## Abstract

**Background:**

Previous studies utilizing self-reported data or polysomnography have found that poor sleep quality is associated with increased risk of morbidity and mortality. However, the association between real-world sleep patterns, including sleep stages, duration, and fragmentation, measured objectively with physiological data and the risk of incident disease among middle-aged and older adults has not yet been systematically evaluated.

**Methods and findings:**

In this cohort study, we analyzed wrist-worn accelerometer data from 95,559 Biobank participants and derived key metrics of real-world sleep patterns: rapid eye movement [REM], N1, N2, and N3; total sleep duration; sleep irregularity; and wakefulness after sleep onset using the SleepNet algorithm. Participants were followed up from the date of accelerometer wear until the first occurrence of disease, death, or April 1, 2024 with median follow-up length of 8.9 years. Phenome-wide association analysis and restricted cubic spline (RCS) analyses were performed using Cox proportional hazard regression, with adjustment for demographic characteristics, lifestyle factors, and environmental exposures, to map an atlas of associations between real-world sleep patterns and the incidence of 1,049 health outcomes. We found that variations in sleep patterns were associated with 156 incidences of diseases. Specifically, higher amount of REM sleep and deep sleep were associated with lower risks of 83 and 7 diseases, respectively, while greater sleep irregularity and increased WASO were linked to elevated risks of 3 and 6 diseases, respectively. RCS analyses revealed significant non-linear relationships between sleep duration and 86 disease phenotypes (*P* for nonlinear <0.05), with the minimum-risk hours for 69 phenotypes predominantly concentrated within the 6–8 hours window. In the category-specific analysis, individuals with extreme short sleep (<5 hours) exhibited the most widespread clinical vulnerabilities, accounting for 37 of the 41 identified significant adverse associations compared to the 6–8 hours reference group. The main limitation of this study was its observational design, which remains susceptible to residual confounding and precludes causal inference.

**Conclusion:**

In summary, this study characterizes real-world sleep patterns and their links to disease risk across multiple systems, highlighting the differential contributions of sleep stages and sleep duration. The findings underscore that maintaining 6–8 hours of sleep is associated with a more favorable sleep architecture and lower disease risk, offering insights for prevention and health promotion.

## Author summary

**Why was this study done?:**

- Self-reported sleep measures often show poor correlation with objective assessments, making it difficult to capture real-world sleep patterns.
- There remains limited understanding of how sleep stages and other real-world sleep patterns relate to health outcomes.
- Large-scale cohort evidence linking objectively measured sleep patterns with incident diseases is still lacking.

**What did the researchers do and find?:**

- We analyzed wrist-worn accelerometer data from 95,559 UK Biobank participants and derived sleep stages and other sleep metrics.
- Phenome-wide association analysis identified 156 significant associations between sleep patterns and incident diseases after Bonferroni correction.
- REM and deep sleep were associated with lower risk of multiple diseases, while sleep irregularity and wakefulness after sleep onset were associated with higher risk.
- Sleep duration exhibited significant non-linear associations with 86 disease phenotypes, with the minimum-risk duration for the majority of these conditions (69 phenotypes) precisely concentrated within a 6–8 hours window.

**What do these findings mean?:**

- These findings highlight that both sleep architecture and sleep duration are associated with disease risk.
- Maintaining 6–8 hours of sleep was associated with more favorable sleep patterns and lower disease risk.
- As this is an observational study, the main limitations are that causality cannot be inferred and residual confounding may remain.

## Abstract 3

In this observational study, Shengzhi Sun and colleagues examine accelerometer-derived sleep patterns in 95,559 participants from the UK Biobank and find that differences in sleep stages, duration, regularity, and fragmentation are associated with the future incidence of a wide range of diseases. The analysis suggests that sleep characterized by higher proportions of REM and deep sleep, along with a typical duration of 6–8 hours, is generally linked to a more favorable health profile, but the observational design does not support causal conclusions.

## 1. Introduction

Sleep is a fundamental human behavior that plays a critical role in overall health and wellbeing. While many previous studies have examined the association between sleep and risks of morbidity and mortality, most have primarily focused on sleep duration as measured by self-reported questionnaires [1,2]. However, self-reported sleep measures often show poor correlation with objective physiological assessments [3–5], making it difficult to accurately capture real-world sleep patterns. Moreover, there remains limited understanding of how other real-world sleep patterns, such as sleep stages (rapid eye movement [REM], N1, N2, and N3) and fragmentation, relate to health outcomes.

The gold standard for objectively assessing sleep patterns is polysomnography (PSG), which provides accurate measurements of real-world sleep stages, including REM, N1, N2, and N3 [6,7]. However, its high cost and technical complexity limit its feasibility for large-scale epidemiological studies. Although consumer-grade wrist-worn devices have popularized sleep monitoring, their sleep staging algorithms are typically proprietary and validated only in small samples, leaving their measurement validity uncertain [8–11]. Furthermore, these devices are predominantly used among younger and middle-aged adults, resulting in a lack of large-scale data in older populations [12]. In contrast, wrist-worn accelerometers have become widely used for objective measurement of sleep patterns in large middle-aged and elderly cohorts, such as nighttime sleep duration, onset, efficiency, and fragmentation [13,14]. A landmark study developed a self-supervised machine learning algorithm, SleepNet, which demonstrated competitive performance in characterizing sleep architecture from wrist-worn accelerometer data [7]. Validated against 1,113 nights of laboratory-based polysomnography recordings, this approach provides a robust methodological framework for deriving sleep stages and sleep regularity metrics from accelerometer signals [7]. Building on these advances, recent studies have leveraged accelerometer-derived sleep features to investigate their associations with cardiometabolic and neurological outcomes [15–17], as well as to explore the genetic architecture of sleep traits through genome-wide association analyses [18]. However, the field still lacks large-scale cohort studies validating and mapping the relationships between accelerometer-derived real-world sleep stages in middle-aged and elderly adults and comprehensive health outcomes.

Examining real-world sleep patterns in middle-aged and older adults is particularly important. First, as people age, they undergo natural changes in sleep architecture, such as reductions in REM and deep sleep and increased nighttime wakefulness [19]. Understanding these changes is crucial for accurately assessing sleep quality and its health implications in older populations. Second, older adults are more susceptible to sleep disorders, like insomnia and sleep apnea, and they often have comorbid conditions that can further complicate real-world sleep patterns and health outcomes [20,21]. Third, as the global population continues to age, it becomes increasingly important to understand the health consequences of sleep stage in older adults.

The UK Biobank is a large, prospective cohort study of over 500,000 participants aged 37–73 years (median age 59 years at baseline) [22]. A subset of more than 100,000 participants provided seven days of wrist-worn triaxial accelerometer data (Axivity AX3) [23], offering an unprecedented opportunity to objectively examine the relationship between accelerometer-derived sleep patterns and incidences of disease outcomes among middle-aged and older adults.

**Fig 1:**
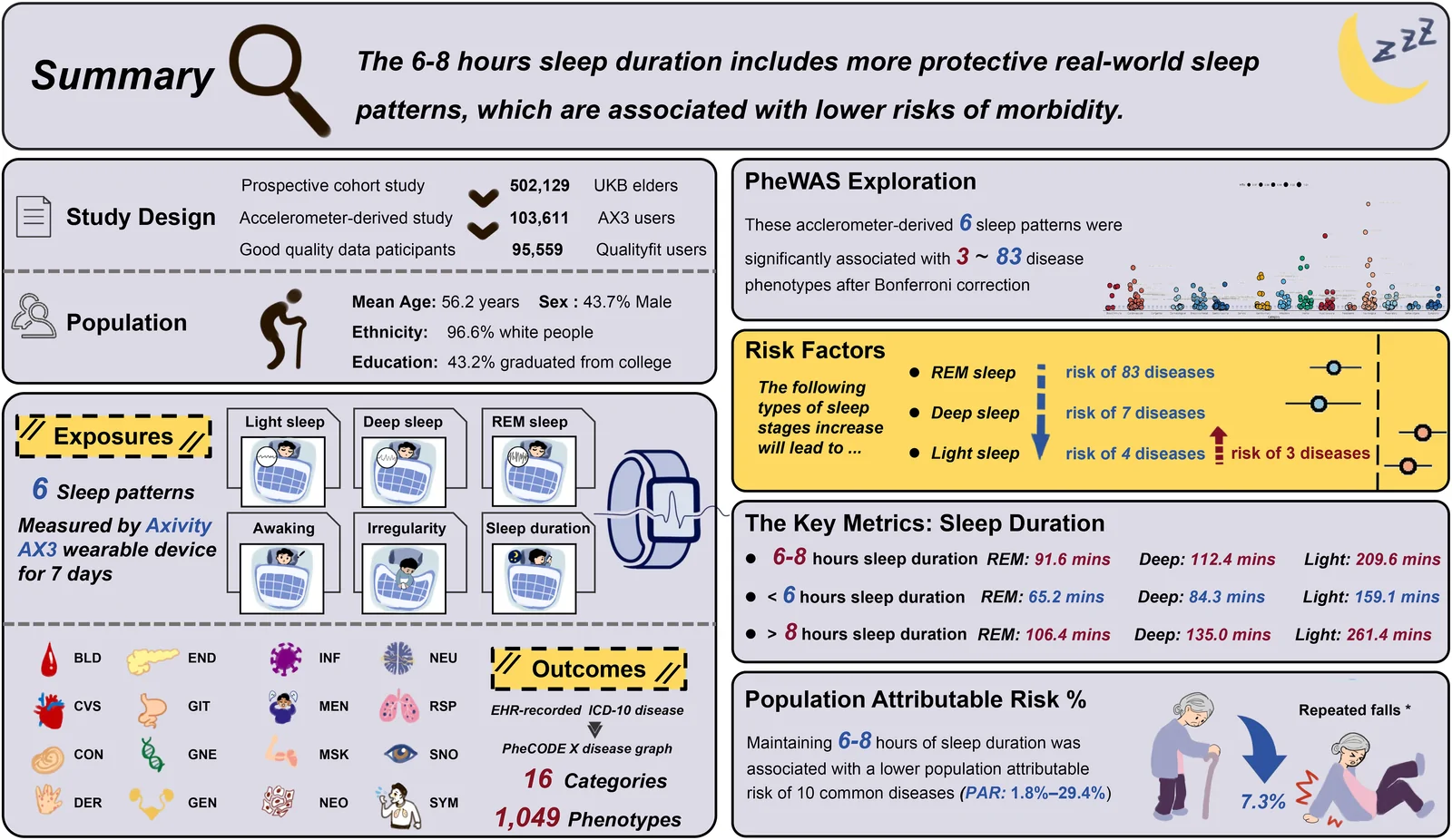
Study summary. The study analyzed wrist-worn accelerometer data from the UK Biobank cohort to map an atlas of associations between real-world sleep patterns and the incidence of 1,049 health outcomes. The top summary box presents the main findings of this study. The left panel shows two vertically arranged methodology boxes: the first details study design and participant characteristics, while the second outlines exposure and outcome variables. Real-world sleep patterns were derived from accelerometer recordings and classified into six sleep patterns using the SleepNet algorithm. Outcomes were incident inpatient diagnoses extracted from electronic health records, coded using the International Classification of Diseases, tenth version (ICD-10), and mapped to 1,049 disease phenotypes by PheCODE Map X. The right panel comprises four results boxes summarized key analyses: a discovery-focused PheWAS, the Cox proportional hazards regression model, ANOVA test, and population attributable risk (PAR). Abbreviations: PheWAS, Phenome-wide association analysis; BLD, Blood/Immune; CSV, Cardiovascular; CON, Congenital; DER, Dermatological; END, Endocrine/Metabolic; GIT, Gastrointestinal; GENE, Genetic; GEN, Genitourinary; INF, Infections; MEN, Mental disorders; NEO, Neoplasms; NEU, Neurological; RSP, Respiratory; SNO, Sense organs; SYM, Symptoms.

In this study (Fig 1), we employed the SleepNet algorithm to classify sleep patterns from the accelerometer data, including sleep stages, duration, and fragmentation. We performed a phenome-wide association study (PheWAS) to systematically estimate the associations between these sleep patterns and 1,049 health outcomes. This approach enables an integrated understanding of how real-world sleep relates to disease risk and provides a systematic foundation for hypothesis generation and prioritization in future research.

## 2. Methods

### 2.1 Study participants

This study used data from the UK Biobank, a large, prospective population-based cohort study. Between 2006 and 2010, 502,129 individuals aged 40–69 years were recruited from 22 assessment centers across England, Scotland, and Wales, representing approximately 5.5% of those invited, and were followed up through 2024 [22]. Cohort profile and data collection have been detailed elsewhere [22]. Ethical approval of the UK Biobank study was granted by the UK National Information Governance Board for Health and Social Care and National Health Service North West Multi-centre Research Ethics Committee (R21/NW/0157) (S1 Table). All participants provided written informed consent before the study. This research was conducted under UK Biobank application number 104745.

Between June 1, 2013 and December 23, 2015, a subset of 236,519 participants was invited to wear a wrist-worn accelerometer for seven consecutive days, of whom 103,611 returned usable data [24]. Participants wore an accelerometer (Axivity Ltd, Newcastle, UK) on their dominant wrist, which recorded raw acceleration at 100 Hz with a dynamic range of ± 8 g. Data were calibrated to local gravity and summarized into 5-s epochs, with each epoch summarized by its mean vector magnitude [24]. Non-wear time was defined as ≥60 min of inactivity with a standard deviation <13.0 mg across all three axes [24].

**Fig 2:**
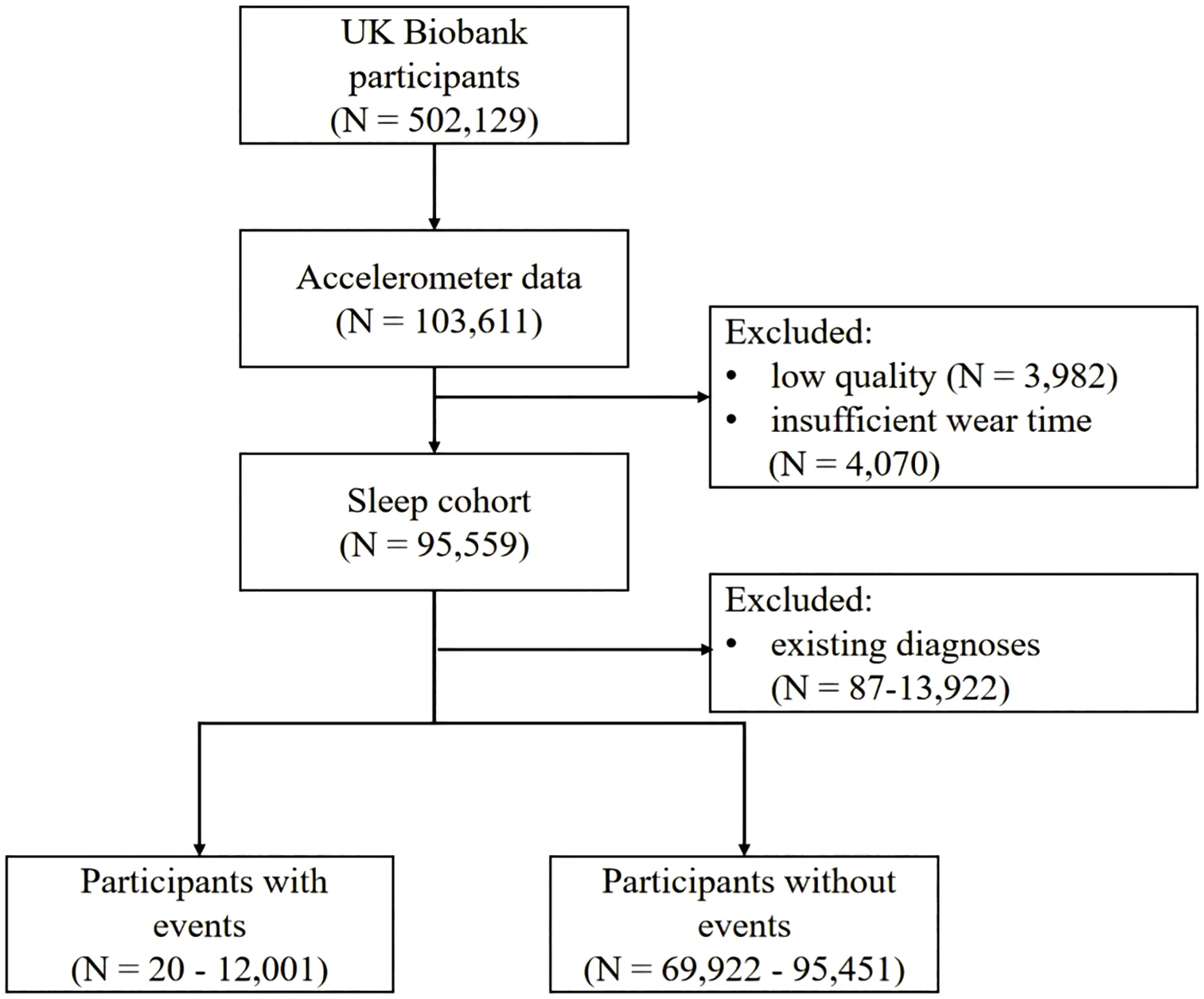
Flow diagram of participants.

We excluded 3,985 participants whose accelerometer data failed quality control (Data-Field 90015), and a further 4,067 participants due to non-wear time exceeding 3 days (Data-Field 90052) or incomplete data across each 1-hour period of the 24-hour cycle (Data-Field 90084). The final analytic sample comprised 95,559 participants (Fig 2).

### 2.2 Accelerometer-derived sleep patterns

Accelerometer data were processed and calibrated using a validated pipeline via the UK Biobank accelerometry toolkit (github.com/OxWearables/biobankAccelerometerAnalysis) [25–27]. Sleep architecture was characterized using SleepNet, a novel deep-learning approach trained on over 1,100 nights of concurrent laboratory-based polysomnography and accelerometry data [7]. The model was further validated across multiple independent cohorts, including the Raine (Gen1 and Gen2), Newcastle, Leicester, and Pennsylvania cohorts, demonstrating its generalizability across diverse populations [7]. To obtain a three-class output, the model aggregated N1, N2, and N3 stages into a single NREM class. For the REM/NREM/wake classification, the model achieved an F1 score of 0.49 (SD 0.10), representing a moderate level of classification performance typical for motion-based sleep staging models. Compared with PSG, the model exhibited mean differences of 48.2 min for sleep duration, −17.1 min for REM duration, 31.1 min for NREM duration, and 9.2% for sleep efficiency. These systematic differences are expected to be non-differential with respect to long-term clinical outcomes, thereby preserving the validity of relative risk rankings in prospective analyses. Nevertheless, as this approach relies on movement-based estimates, it may not fully capture the biological nuances of sleep stages defined by gold-standard PSG. Although this approach demonstrates improved performance over traditional baseline algorithms based on hand-crafted features, its agreement with gold-standard PSG measurements remains relatively modest [7,28,29]. Consequently, the sleep stage characteristics derived in this study represent movement-based estimates and may not fully correspond to the biological sleep stages defined by PSG. For the primary analysis, six sleep parameters were derived: total sleep duration (total sleep time per 24-hour noon-to-noon interval), REM sleep duration, NREM sleep duration (comprising light sleep [N1 + N2] and deep sleep [N3]), wake after sleep onset (WASO), and sleep irregularity (defined as the standard deviation [SD] of daily sleep duration) [12].

### 2.3 Study outcomes

The primary outcomes were incident inpatient diagnoses, ascertained through linkage to electronic health records and coded using the International Classification of Diseases, Tenth Revision (ICD-10). For each participant, the baseline of follow-up was defined as the date of their first accelerometer wear. To ensure that outcomes were genuinely incident, we adopted a phenotype-specific framework for disease history ascertainment and risk set construction. For each disease phenotype, participants with a recorded diagnosis of that specific condition prior to baseline were considered not at risk and were therefore excluded from the corresponding risk set (left truncation based on available historical records). Notably, participants were not excluded entirely if they had a prior diagnosis of a different phenotype; thus, the same individual could contribute to the risk set for one outcome while being excluded from another. To minimize reverse causation, incident cases occurring within the first six months after accelerometer monitoring were excluded.

All 10,515 unique ICD-10 codes recorded in inpatient data were aggregated and mapped to 2,050 distinct clinical phenotypes using the PheCODE Map X system. This validated schema reduces collinearity and multiple testing burden by consolidating highly correlated or synonymous codes into clinically coherent disease entities [30–32]. To ensure statistical robustness and minimize outcome misclassification, two additional exclusion criteria were applied to these 2,050 phenotypes. First, we excluded phenotypes designated as “nonspecific”, “Other,” “Unspecified,” (e.g., “Other specified viral infections”). Second, we excluded phenotypes with fewer than 20 incident cases to ensure convergence of regression models and stable estimation of hazard ratios in the presence of sparse data. After applying these sequential filters, 1,049 well-defined phenotypes were retained for final analysis.

### 2.4 Covariates

Demographic and lifestyle covariates were collected using standard and structured questionnaires [33] at the same baseline visit (2006–2010) and accelerometer wear (2013–2015). Covariates were chosen a priori based on established associations with both sleep and chronic disease [34,35]. Demographic variables included age at enrollment (years), sex (male and female), Townsend deprivation index (TDI), ethnicity (White, Asian, Black, Mixed, and Other), educational attainment (high/degree, upper secondary, lower secondary/vocational, and none), employment status (employed, retired, unable to work due to health reasons, unemployed in education, and looking after home or unpaid), body mass index (BMI), and urbanicity (urban versus rural living). Lifestyle factors comprised smoking status (current, previous, and never), alcohol consumption (daily, three or four times per week, once or twice per week, one to three times per month, special occasions only, and never), physical activity (MET-minutes), time spent watching TV (hours), time spent using a computer (hours), and mobile phone use duration (never used mobile phone at least once per week, one year or less, two to four years, five to eight years, and more than eight years). Environmental exposures included outdoor particulate matter with an aerodynamic diameter ≤2.5 μm (PM_2.5_), generated using a Land Use Regression (LUR) model, and the average sound pressure level LAeq over the night period from 23:00 to 07:00. Detailed definitions and coding for all covariates were provided in S2 Table.

### 2.5 Statistical analysis

Participant characteristics were summarized using descriptive statistics: sleep patterns were reported as medians with interquartile ranges (IQR) and ranges; continuous variables as means and SDs; and categorical variables as counts and percentages.

We used Cox proportional hazards regression to estimate associations between accelerometer-derived sleep metrics and each incident disease phenotypes. Follow-up time was used as the underlying time-scale, defined from the date of accelerometer wear to the earliest of first occurrence of the disease of interest, date of death, or April 1, 2024. Models were adjusted for age, sex, TDI, ethnicity, educational attainment, employment status, BMI, and urbanicity, smoking status, alcohol consumption, physical activity, time spent watching TV, time spent using a computer, and mobile phone use duration, residential particulate matter, and nighttime noise levels. Results were reported as hazard ratios (HRs) per IQR increase in each sleep parameters. Statistical significance was initially assessed using a false discovery rate (FDR) threshold of *q* < 0.05, followed by a Bonferroni-corrected threshold of *α* < 0.05/1,049 = 4.76 × 10^−5^ to account for multiple testing. Given previous evidence of U-shaped associations between sleep duration and incident disease [12,36–38], we conducted non-linear analyses for all phenotypes. Restricted cubic spline analyses were conducted to assess potential nonlinear relationships, with knots placed at the 5th, 27.5th, 50th, 72.5th, and 95th percentiles of each sleep parameter distribution [39]. We used Wald *χ*^2^ tests to assess deviations from linearity [12,39,40]. To reduce the influence of sparse observations at the extremes, the estimated risk nadir was identified as the sleep duration corresponding to the minimum predicted hazard ratio within the 5th-95th percentile range of the observed distribution. When the minimum occurred at either boundary of this range, it was classified as a boundary minimum rather than a clearly identified internal nadir.

According to the minimum risk interval identified through non-linear analysis, sleep duration was categorized into three groups: the low-risk reference interval, short sleep duration (shorter than the reference interval), and long sleep duration (longer than the reference interval). To investigate potential variations in sleep architecture (light, deep, and REM sleep) across these intervals, we performed ANOVA followed by Tukey’s post-hoc tests. Additionally, Cox proportional hazards regression models were constructed, adjusting for the aforementioned covariates, to estimate HRs for short and long sleep durations relative to the reference group. To obtain a more granular comparison of risk differences, sleep duration was further stratified into five distinct categories based on the morphology of the RCS curves. For each category, we estimated the HR and the population attributable risk percent (PAR%) relative to the low-risk reference group. PAR was estimated by *p* (HR − 1)/HR [41,42], where *p* represents the prevalence of each specific sleep category, and HR denotes the cause-specific morbidity risk for that category compared to the reference group.

We conducted several sensitivity analyses to confirm the robustness of our findings. First, to assess the influence of sleep disorder, we further adjusted for the diagnosis history of sleep-related phenotypes, including sleep disorders, insomnia, hypersomnia, circadian rhythm sleep disorders, parasomnias, sleepwalking, and narcolepsy. Second, to evaluate the potential impact of season variation, we further adjusted the models for the season recorded by accelerometer timestamps. Third, to further isolate the independent effects of sleep irregularity on incident diseases, analyses of sleep irregularity were additionally adjusted for daily total sleep duration. Fourth, to minimize the impact of subclinical disease at baseline, we expanded the exclusion window for early incident cases from 180 days to 2 years following the accelerometry assessment. Fifth, to assess whether observed associations were driven by underlying chronic conditions, we implemented a comorbidity-adjusted model. This model controlled for pain, chronic pain, major depressive disorder, anxiety disorders, hypertension, type 2 diabetes, and various types of illicit drug use (alcohol, opioids, cannabis, and sedatives/hypnotics). Finally, to investigate whether sleep staging associations are independent of total sleep duration, we utilized compositional metrics by calculating the proportion of each sleep stage (REM, Deep, and Light sleep) relative to total sleep duration. This approach allows for a nuanced exploration of how relative sleep composition impacts disease phenotypes.

This exploratory, hypothesis-generating PheWAS examined whether accelerometer-derived sleep metrics were associated with incident disease phenotypes and whether these associations were nonlinear. The analyses performed included Cox proportional hazards regression across 1,049 disease phenotypes, correction for multiple testing, RCS analysis, and sensitivity analyses. This study did not have a prospectively registered protocol or formal prospective analysis plan. During peer review, the nonlinear analyses were expanded from phenotypes showing significant associations in the primary Cox analyses to all 1,049 phenotypes to avoid significance-based selection and provide a more comprehensive assessment of potential nonlinear associations. Additional sensitivity analyses were conducted to assess the influence of pre-existing sleep disorders, exclusion of incident cases occurring within two years after accelerometry assessment, baseline comorbidities, and sleep-stage composition relative to total sleep duration. These modifications were made in response to peer review and were not prompted by unanticipated patterns in the data. Statistical analyses were performed in R (v.4.2.2, R Project https://www.r-project.org) and Python 3.8 on the UKB-RAP cloud-based platform. Specifically, the survival package [43] was used to construct Cox proportional hazards models and estimate hazard ratios. The rms package [44] was employed for RCS analyses and Wald *χ*^2^ tests to evaluate non-linearity. For multiple testing corrections, the stats package [45] was used to calculate FDR-adjusted *q*-values. Data manipulation and visualization were primarily supported by the tidyverse suite [46], including dplyr and ggplot2 packages. This study is reported as per the Strengthening the Reporting of Observational Studies in Epidemiology (STROBE) guideline and the Reporting of Studies Conducted using Observational Routinely-Collected Data (RECORD) guideline (S1 Checklist).

## 3. Results

### 3.1 Study population

**Fig 3:**
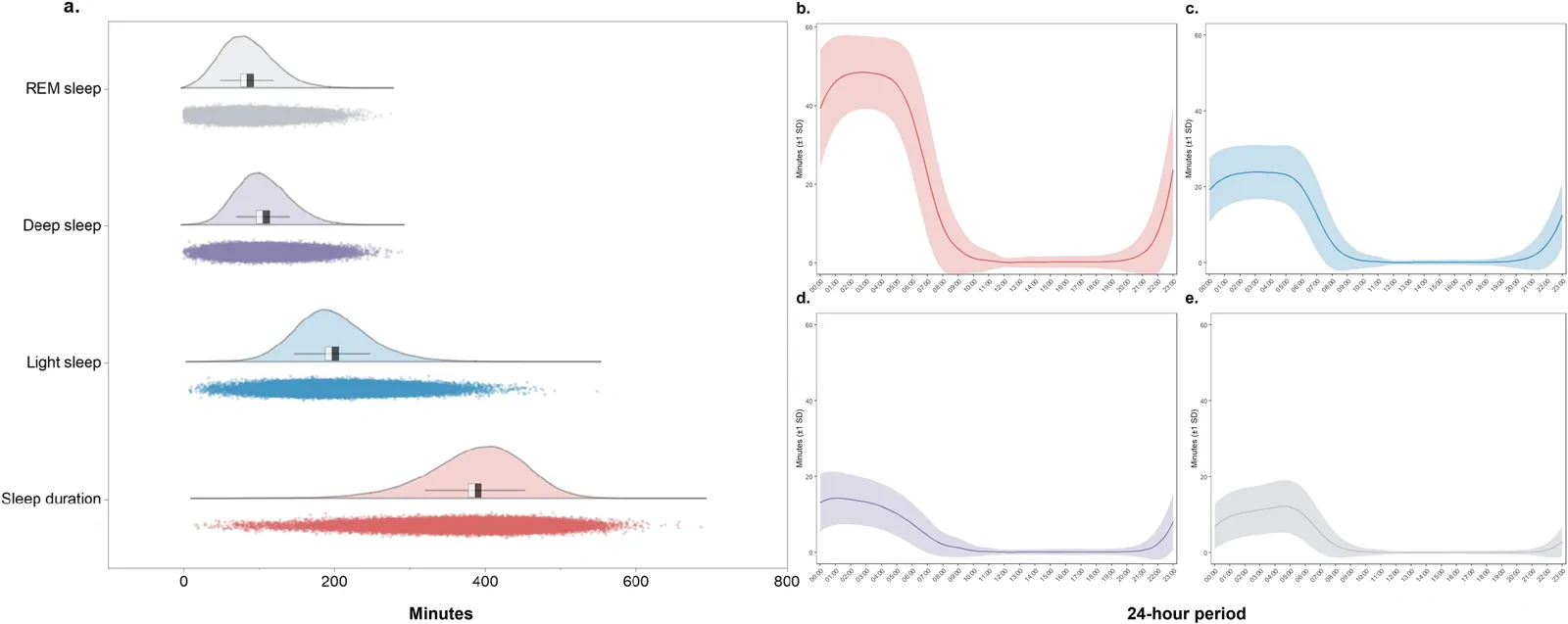
Distribution of accelerometer-derived sleep metrics and 24-hour sleep traces in middle-aged and older adults. Panel **a** illustrates the distribution of overall accelerometer-derived sleep stages and total sleep duration among older adults in the UK Biobank. Panels **b** through **e** display 24-hour traces of individual sleep metrics: (**b**) total sleep duration, (**c**) light sleep stage, (**d**) deep sleep stage, and **(e)** REM sleep stage, all derived from accelerometer recordings.

Of 502,129 UK Biobank participants, 95,559 individuals with complete accelerometer-derived sleep data were included (Fig 2). In this study, participants were followed from first time of accelerometer wear with median follow-up duration of 8.9 years. The participants’ characteristics at baseline including mean age were 56.2 years (standard deviation 7.8); 43.7% were male, and 96.9% identified as white. The majority were employed (61.9%) and 43.4% held a college or university degree (**Table 1**). Most participants had sleep onset between 9 and 11 pm. The median total sleep duration daily was 392.4 min (interquartile range [347.9,431.8]); median sleep irregularity and WASO were 73.5 min (interquartile range [46.2,139.2]) and 64.1 min (interquartile range [45.4,89.0]), respectively. Median durations for sleep stages were 81.7 min (interquartile range [59.1,106.6]) for REM, 102.6 min (interquartile range [80.1,127.6]) for deep sleep, and 193.3 min (interquartile range [163.1,226.9]) for light sleep (Fig 3).

**Table 1:** Baseline characteristics of study participants.

Characteristics	All participants (*N* = 95,559)
Age, years	
Mean (SD)	56.2 (7.8)
Sex, *n* (%)	
Male	41,743 (43.7)
Female	53,816 (56.3)
Townsend deprivation index, *n* (%)	
Q1	24,110 (25.2)
Q2	24,003 (25.1)
Q3	23,804 (24.9)
Q4	23,642 (24.7)
Ethnicity, white, *n* (%)	
White	92,632 (96.9)
Black	1,102 (1.2)
Asian	800 (0.8)
Mixed	518 (0.5)
Other	507 (0.5)
Education, *n* (%)	
Higher/Degree	41,484 (43.4)
Upper secondary	12,608 (13.2)
Lower secondary/Vocational	33,433 (35.0)
None	8,034 (8.4)
Employment status, employed, *n* (%)	
Employed	59,188 (61.9)
Retired	29,839 (31.2)
Unable to work for health reason	1,999 (2.1)
Unemployed	1,173 (1.2)
In education	316 (0.3)
Looking after home or unpaid	3,044 (3.2)
Urbanicity, *n* (%)	
Urban	80,538 (84.3)
Rural/Town	15,021 (15.7)
BMI, kg/m^2^	
Mean (SD)	26.7 (4.5)
Smoking, *n* (%)	
Current	54,585 (57.1)
Previous	34,359 (36.0)
Never	6,615 (6.9)
Alcohol, *n* (%)	
Daily	21,848 (22.9)
Three or four times a week	24,845 (26.0)
Once or twice a week	23,968 (25.1)
One to three times a month	10,396 (10.9)
Special occasions only	9,084 (9.5)
Never	5,418 (5.7)
Weekly physical activity, MET-minutes	
Mean (SD)	2,517.1 (2,435.7)
Watching TV, hours	
Mean (SD)	2.5 (1.5)
Computer using, hours	
Mean (SD)	1.3 (1.4)
Phone using, *n* (%)	
Never used mobile phone at least once per week	13,934 (14.6)
One year or less	1,893 (2.0)
Two to four years	14,545 (15.2)
Five to eight years	29,865 (31.3)
More than eight years	35,322 (37.0)
PM_2.5_, μg/m^3^	
Mean (SD)	9.9 (1.0)
Nighttime noise, dB	
Mean (SD)	46.5 (4.2)

Abbreviations: SD, standard deviation; IQR, interquartile range; BMI, body mass index.

### 3.2 Phenome-wide association analysis of sleep patterns

**Fig 4:**
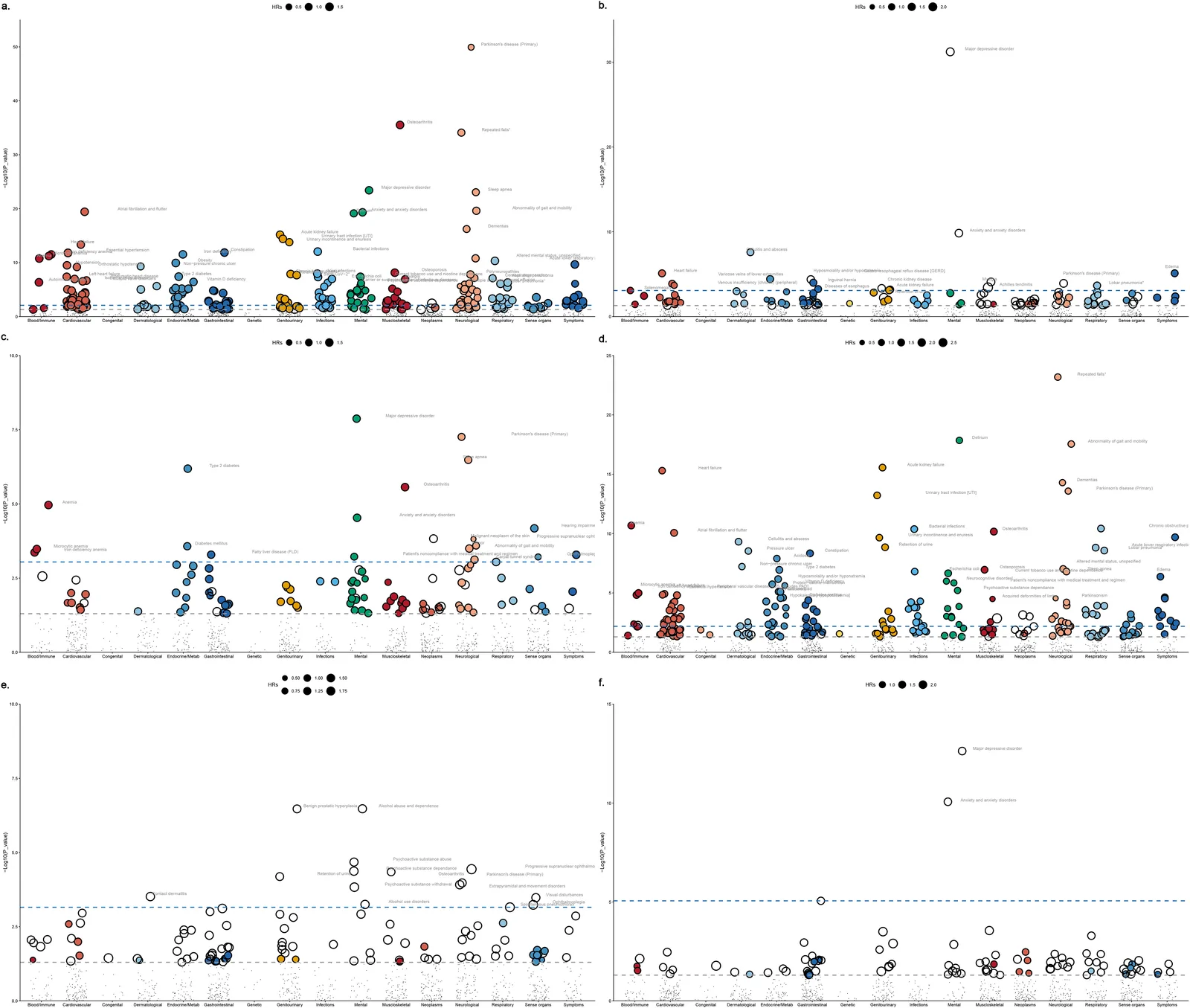
Phenome-wide association analysis of real-world sleep patterns with incident diseases. Phenome-wide association analysis was performed to explore the associations between accelerometer-derived sleep metrics and incident diseases. Results were estimated from the fully adjusted models. The false discovery rate significance threshold (*q* < 0.05) is indicated by a blue line; the nominal *p* threshold of 0.05 is shown as a gray line. All reported *P* values are two-tailed. Filled circles represent phenotypes with hazard ratios <1 and *p* < 0.05; open circles represent hazard ratios >1 and *p* < 0.05. Phenotypes with *p* > 0.05 are indicated by faint points below the gray line. P*-*values were calculated using two-sided Wald tests from Cox proportional hazards regression models. **(a)** REM sleep stage; **(b)** light sleep stage; **(c)** deep sleep stage; **(d)** total sleep duration; **(e)** wakefulness after sleep onset; **(f)** sleep irregularity.

**Fig 5:**
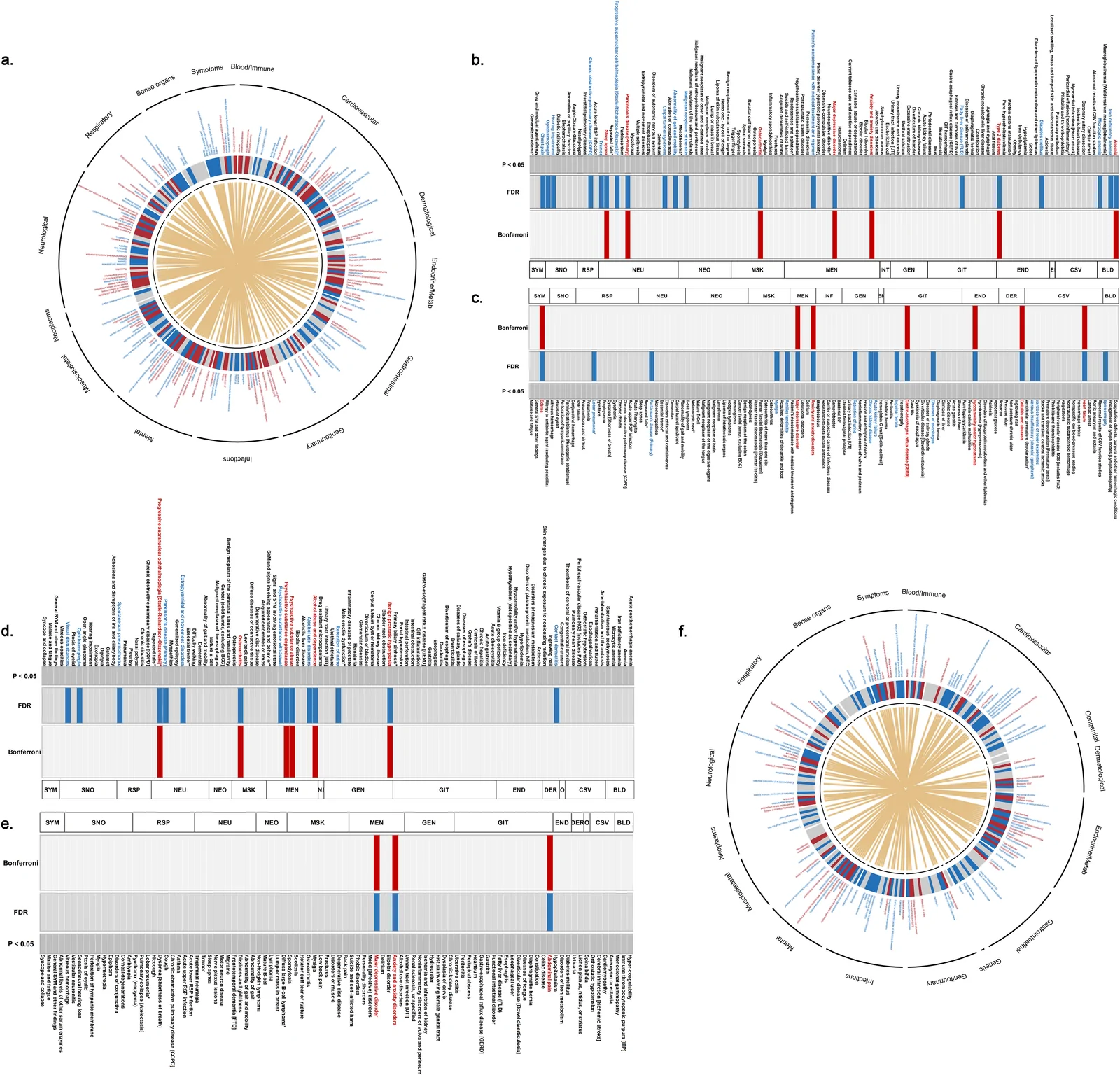
Systematic significance correction for the association between accelerometer-derived sleep metrics and incident disease. All phenotypes shown in the panels represent nominally significant (*p* < 0.05; two-sided Wald tests from Cox proportional hazards regression models) associations with real-world sleep patterns. The outermost ring of panels **(a)** and **(f)** represents disease categories associated with REM sleep and total sleep duration, respectively. The second ring details specific phenotypes. Phenotypes that passed false discovery rate (FDR) correction are shown in blue, while those meeting the Bonferroni correction threshold are highlighted in red. The third ring clusters all phenotypes that passed either significance correction. Panels (**b–e)** show significance corrections for deep sleep, light sleep, wakefulness after sleep onset, and sleep irregularity. In these, phenotypes passing FDR correction are marked with a blue box; those also Bonferroni-significant are shown in red.

A PheWAS evaluating 1,049 incident health outcomes identified 370 associations (false discovery rate <0.05) with accelerometer-derived sleep patterns: 212 for sleep stages, 140 for total sleep duration, 15 for WASO, and 3 for sleep irregularity (Fig 4). After Bonferroni correction (*α* = 0.05/1049), 97 associations for sleep stages (83 for REM, 7 for deep, and 7 for light), 50 for total sleep duration, 6 for WASO, and all 3 for sleep irregularity remained significant (Fig 5 and S3–S4 Tables).

### 3.3 Sleep stages and disease risk

**Fig 6:**
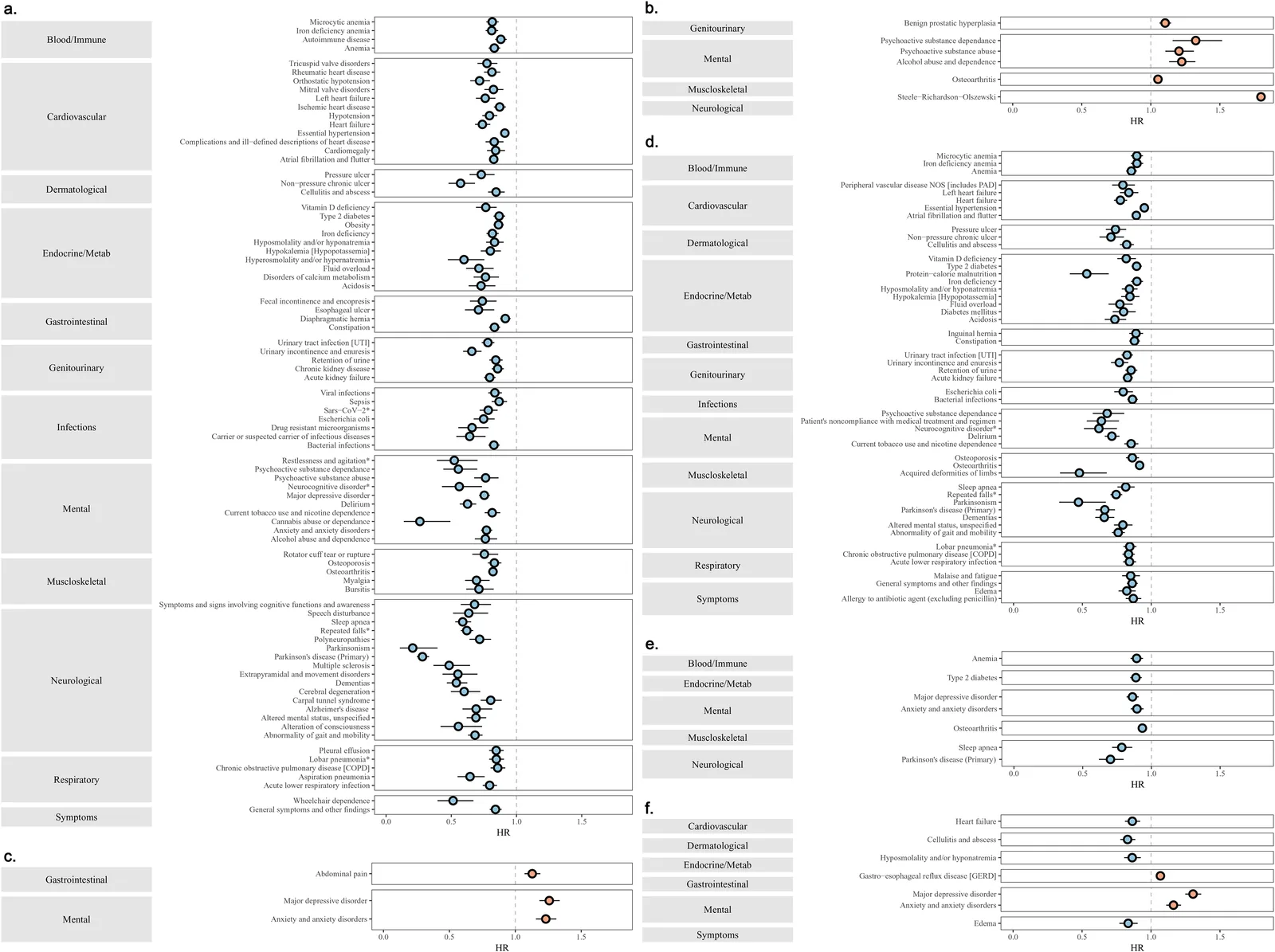
Forest plots for Bonferroni-significant associations. Forest plots display systematically Bonferroni-significant associations between accelerometer-derived sleep metrics and incident disease. HRs and 95% CI were calculated comparing the 25th percentile with 75th percentile of each sleep metrics. Boxes represent HR estimates, and horizontal dashed lines represent 95% CIs. Blue dots indicate significant negative associations; orange indicate positive associations. Panels: **(a)** REM sleep stage; **(b)** wakefulness after sleep onset; **(c)** sleep irregularity; **(d)** total sleep duration; **(e)** deep sleep stage; **(f)** light sleep stage. Abbreviations: HR, Hazard ratios; CI, Confidence interval.

Increased REM sleep duration (per interquartile range, 47.6 min) was associated with lower risk of 83 incident diseases across 12 categories, including heart failure (hazard ratio 0.74, 95% confidence interval [0.68,0.80]; *p* < 0.001), hypotension (hazard ratio 0.79, 95% confidence interval [0.74,0.85]; *p* < 0.001), atrial fibrillation (hazard ratio 0.83, 95% confidence interval [0.79,0.86]; *p* < 0.001), ischemic heart disease (hazard ratio 0.87, 95% confidence interval [0.83,0.92]; *p* < 0.001), parkinsonism (hazard ratio 0.20, 95% confidence interval [0.11,0.39]; *p* < 0.001), multiple sclerosis (hazard ratio 0.48, 95% confidence interval [0.36,0.64]; *p* < 0.001), dementias (hazard ratio 0.54, 95% confidence interval [0.47,0.62]; *p* < 0.001), and Alzheimer’s diseases (hazard ratio 0.69, 95% confidence interval [0.57,0.81]; *p* < 0.001) (Fig 6A and S3 Table). Increased deep sleep duration (per interquartile range, 47.5 min) was associated with lower risk of 7 diseases across 5 categories, including type 2 diabetes (hazard ratio 0.89, 95% confidence interval [0.85,0.93]; *p* < 0.001), major depressive disorder (hazard ratio 0.86, 95% confidence interval [0.82,0.91]; *p* < 0.001), sleep apnea (hazard ratio 0.78, 95% confidence interval [0.71,0.86]; *p* < 0.001), and Parkinson’s diseases (hazard ratio 0.70, 95% confidence interval: [0.62,0.80]; *p* < 0.001) (Fig 6E and S3 Table). Greater light sleep duration was associated with higher risks for conditions such as major depressive disorder (hazard ratio 1.31, 95% confidence interval [1.25,1.36]; *p* < 0.001), but lower risks for heart failure (hazard ratio 0.86, 95% confidence interval [0.81,0.92]; *p* < 0.001) and cellulitis (hazard ratio 0.83, 95% confidence interval [0.78,0.89]; *p* < 0.001) (Fig 6F and S3 Table).

### 3.4 Sleep regularity, WASO, and disease risk

Higher WASO (per interquartile range, 43.6 min) was associated with elevated risk of 6 diseases, including psychoactive substance dependence (hazard ratio 1.33, 95% confidence interval [1.16,1.52]; *p* < 0.001), alcohol abuse (hazard ratio 1.22, 95% confidence interval [1.13,1.32]; *p* < 0.001), and osteoarthritis (hazard ratio 1.05, 95% confidence interval [1.03,1.08]; *p* < 0.001) (Fig 6B and S3 Table). Increased sleep irregularity (per interquartile range, 93.0 min) was linked to higher risk of abdominal pain (hazard ratio 1.13, 95% confidence interval [1.07,1.19]; *p* < 0.001), anxiety and anxiety disorders (hazard ratio 1.23, 95% confidence interval [1.16,1.31]; *p* < 0.001), and major depressive disorder (hazard ratio 1.26, 95% confidence interval [1.18,1.34]; *p* < 0.001) (Fig 6C and S3 Table).

### 3.5 Total sleep duration and disease risk

Longer total sleep duration (per interquartile range, 83.9 min) was generally associated with lower risk of 50 conditions (Fig 6D and S3 Table), particularly among endocrine diseases, such as protein-calorie malnutrition (hazard ratio 0.53, 95% confidence interval [0.41,0.69]; *p* < 0.001), acidosis (hazard ratio 0.74, 95% confidence interval [0.66,0.82]; *p* < 0.001), and type 2 diabetes (hazard ratio 0.89, 95% confidence interval [0.86,0.93]; *p* < 0.001).

### 3.6 Nonlinear relationship between sleep duration and incident diseases

**Fig 7:**
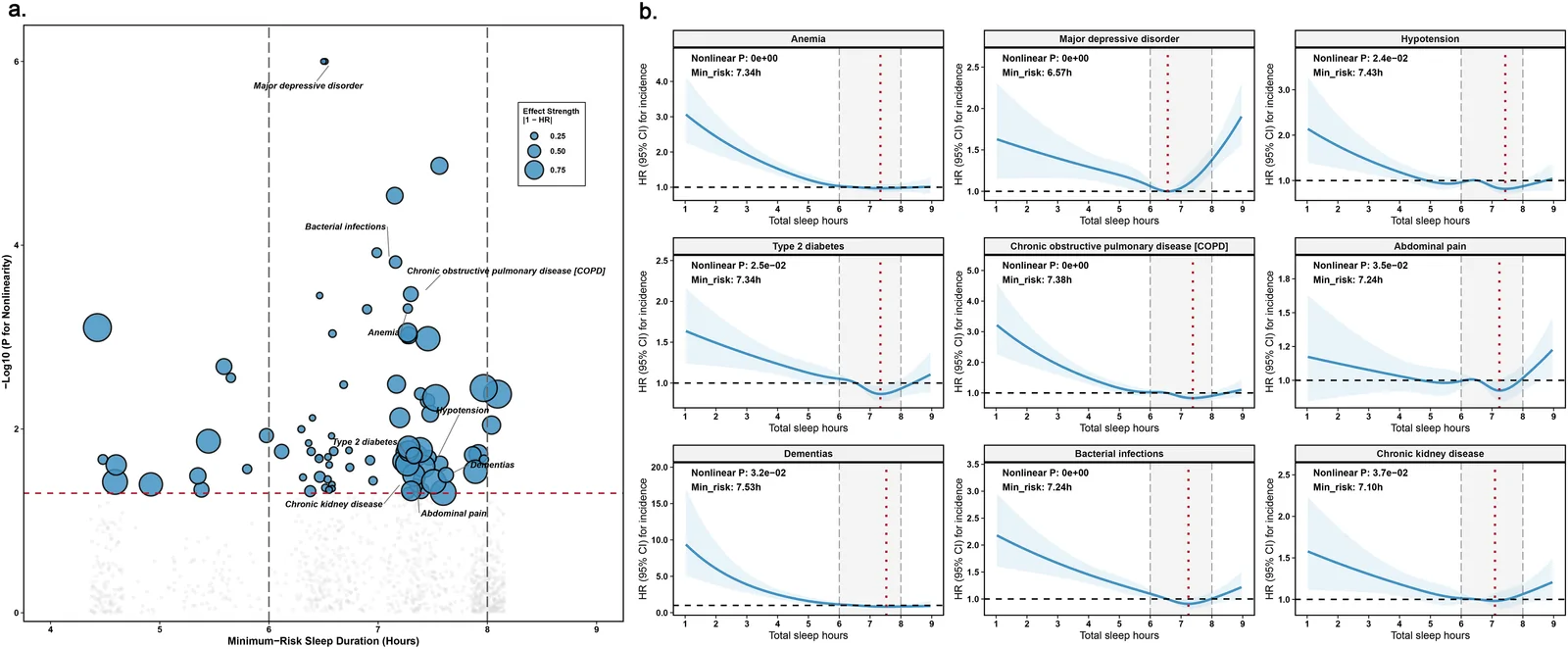
Nonlinear association between sleep duration and incident diseases. **(a)** Overview of the minimum-risk sleep duration across all analyzed disease phenotypes. The x-axis represents the sleep duration associated with the lowest disease risk. The y-axis shows the statistical significance of non-linearity as −log^10^ (*P* for nonlinear). Statistically significant non-linear associations (*P* for nonlinear < 0.05) are highlighted as blue bubbles above the horizontal red dashed line, with the bubble size corresponding to the effect strength (|1 − HR|) of the association. Non-significant phenotypes are displayed as small gray dots clustered below the significance threshold line. Vertical dashed lines delineate the 6 to 8 hours optimal sleep window. **(b)** Restricted cubic splines analysis examined nonlinear relationships between total sleep duration and incident disease, with knots at the 5th, 27.5th, 50th, 72.5th, and 95th percentiles of the sleep duration distribution. The Wald *χ*^2^ tests (*p* < 0.05) indicate significant nonlinearity.

To exhaustively investigate potential non-linear relationships between total sleep duration and incident disease, RCS analyses were performed across all 1,049 phenotypes. A total of 86 phenotypes demonstrated statistically significant non-linear associations with incident diseases (*P* for nonlinear <0.05). Among these, we observed that the minimum-risk hours for 69 disease phenotypes were predominantly concentrated within 6–8 hours sleep duration window, specifically including anemia (7.34 hours), major depressive disorder (6.57 hours), hypotension (7.43 hours), type 2 diabetes (7.34 hours), chronic obstructive pulmonary disease (7.38 hours), abdominal pain (7.24 hours), dementias (7.53 hours), bacterial infections (7.24 hours), and chronic kidney disease (7.10 hours) (Fig 7). In contrast, 12 phenotypes exhibited risk nadirs between 4 and 6 hours of sleep duration, whereas 5 phenotypes, the lowest predicted risk occurred near the upper boundary of the prespecified range. Detailed phenotype-specific risk nadirs are presented in S5 Table.

### 3.7 Adults sleeping 6–8 hours versus other durations

As the relationship between sleep duration and incident diseases might be nonlinear, we categorized participants into three groups: <6 hours, 6–8 hours (reference group), and >8 hours. Older adults sleeping 6–8 hours exhibited significantly longer absolute durations of REM, deep, and light sleep compared to those sleeping <6 hours, though these durations were naturally longest in the >8 hours group (all *p* < 0.001). Notably, regarding sleep architecture proportions, the 6–8 hours group showed the highest REM sleep proportion (0.222 ± 0.081). While the ANOVA suggests significant across-group differences in deep and light sleep proportions (all *p* < 0.001), the 6–8 hours group actually maintained a stable deep sleep proportion (27.2%) identical to the short-sleep group, and a lower light sleep proportion (50.7%) compared to the long-sleep group (51.9%). Furthermore, this group demonstrated markedly lower sleep irregularity and WASO proportions compared to short sleepers (**Table 2**).

**Table 2:** Sleep pattern differences in older adults sleeping 6–8 hours vs. other durations.

Variable	<6 hours	6–8 hours	>8 hours	*P* for ANOVA
REM sleep (min)	65.242 (27.895)	91.628 (34.217)	106.421 (40.279)	<0.001
Deep sleep (min)	84.275 (28.982)	112.395 (32.971)	134.989 (37.699)	<0.001
Light sleep (min)	159.133 (39.229)	209.572 (42.727)	261.426 (53.662)	<0.001
Sleep irregularity	124.039 (51.697)	78.845 (49.941)	57.889 (34.716)	<0.001
Wake after sleep onset	73.711 (42.33)	70.159 (33.551)	69.146 (34.553)	<0.001
REM sleep proportion	0.210 (0.082)	0.222 (0.081)	0.212 (0.080)	<0.001
Deep sleep proportion	0.273 (0.085)	0.272 (0.077)	0.269 (0.075)	<0.001
Light sleep proportion	0.517 (0.104)	0.507 (0.096)	0.519 (0.102)	<0.001
Sleep irregularity proportion	0.422 (0.218)	0.194 (0.129)	0.115 (0.068)	<0.001
Wake after sleep onset proportion	0.248 (0.205)	0.171 (0.084)	0.137 (0.068)	<0.001

All the proportion of sleep metrics are calculated relative to total sleep duration.

Abbreviations: REM, rapid eye movement; SD, standard deviation.

Compared with those who maintained 6–8 hours’ sleep duration, those sleeping outside this range was associated with higher risk of 55 incident diseases after FDR correction. Of these, 51 phenotypes were observed in participants sleeping less than 6 hours, and 4 in those sleeping more than 8 hours. After Bonferroni correction, 24 disease associations remained significant for participants with less than 6 hours of sleep, including heart failure (hazard ratio 1.30, 95% CI [1.16,1.45]; *p* < 0.001), type 2 diabetes (hazard ratio 1.18, 95% confidence interval [1.09,1.27]; *p* < 0.001), and Chronic obstructive pulmonary disease (hazard ratio 1.24, 95% confidence interval [1.13,1.37]; *p* < 0.001). For individuals sleeping more than 8 hours compared to 6–8 hours, only major depressive disorder remained significant (hazard ratio 1.60, 95% confidence interval [1.39,1.84]; *p* < 0.001) (S6 Table).

In the granular five-category analysis of sleep duration (<5 hours, 5–6 hours, 6–8 hours, 8–9 hours, and ≥9 hours), we identified 41 significant associations with higher disease risks compared to the 6–8 hours reference group after Bonferroni correction. Notably, 37 of these associations were observed in the group with sleep duration of less than 5 hours. Furthermore, 2 phenotypes exhibited consistently higher risks in both the <5 hours and 5–6 hours groups compared to the 6–8 hours reference, including repeated falls, and lobar pneumonia. Regarding longer sleep durations, in the comparison between the 8 and 9 hours group and the reference, major depressive disorder remained a significant higher risk (hazard ratio 1.55, 95% confidence interval [1.34,1.78]; *p* < 0.001) and (population attributable risk percent 2.63, 95% confidence interval [1.89,3.27]). Additionally, in the extreme long sleep group (>9 hours), a significant association was noted for Bipolar I disorder; however, this estimate was accompanied by a notably wide confidence interval due to the limited number of incident events in this specific stratum (Table 3).

**Table 3:** Multivariable-adjusted HR (95% CI) and PAR (95% CI) for sleep duration in relation to specific diseases.

Phenotypes	Comparison	Events	HR [95% CI]	PAR% [95% CI]
Parkinsonism	<5 h	31	5.86 [2.57, 13.37]	29.43 [21.67, 32.83]
Alcohol use disorders	<5 h	148	2.68 [1.76, 4.08]	14.82 [10.23, 17.85]
Wheelchair dependence	<5 h	140	2.49 [1.62, 3.82]	14.51 [9.26, 17.93]
Non-pressure chronic ulcer	<5 h	307	2.31 [1.72, 3.10]	12.93 [9.57, 15.44]
Dementias	<5 h	458	2.57 [2.00, 3.31]	11.62 [9.51, 13.26]
Panic disorder [episodic paroxysmal anxiety]	<5 h	196	2.29 [1.57, 3.35]	11.22 [7.21, 13.96]
Pressure ulcer	<5 h	512	2.02 [1.60, 2.55]	10.46 [7.77, 12.59]
Parkinson’s disease (Primary)	<5 h	428	2.37 [1.82, 3.09]	10.27 [8.02, 12.00]
Delirium	<5 h	899	2.07 [1.73, 2.48]	9.54 [7.77, 11.02]
Acidosis	<5 h	442	1.90 [1.47, 2.45]	9.22 [6.24, 11.52]
Symptoms and signs involving cognitive functions and awareness	<5 h	303	2.11 [1.53, 2.91]	8.68 [5.68, 10.84]
Bipolar I disorder	>9 h	23	21.44 [5.03, 91.45]	8.29 [6.97, 8.60]
Abnormality of gait and mobility	<5 h	1,357	1.85 [1.59, 2.16]	7.98 [6.45, 9.29]
Peripheral vascular disease NOS [includes PAD]	<5 h	486	1.78 [1.39, 2.29]	7.57 [4.81, 9.72]
Repeated falls*	<5 h	1,591	1.83 [1.59, 2.11]	7.32 [5.97, 8.50]
Urinary incontinence and enuresis	<5 h	797	1.77 [1.45, 2.17]	7.28 [5.20, 8.99]
Heart failure	<5 h	1,397	1.73 [1.49, 2.01]	7.12 [5.55, 8.46]
Sleep apnea	<5 h	867	1.52 [1.26, 1.84]	6.51 [3.95, 8.62]
*Escherichia coli*	<5 h	757	1.68 [1.36, 2.07]	6.19 [4.04, 7.93]
Vitamin D deficiency	<5 h	808	1.60 [1.31, 1.96]	6.10 [3.83, 7.94]
Repeated falls*	5–6 h	1,591	1.33 [1.18, 1.50]	5.91 [3.59, 7.95]
Acute lower respiratory infection	<5 h	1,776	1.57 [1.36, 1.80]	5.49 [4.06, 6.74]
Cellulitis and abscess	<5 h	1,444	1.51 [1.30, 1.76]	5.24 [3.56, 6.69]
Chronic obstructive pulmonary disease [COPD]	<5 h	1,916	1.50 [1.31, 1.71]	5.12 [3.65, 6.40]
Edema	<5 h	948	1.49 [1.23, 1.79]	5.00 [2.86, 6.76]
Lobar pneumonia*	5–6 h	1,818	1.26 [1.13, 1.42]	4.99 [2.73, 7.00]
Sars-CoV-2*	<5 h	1,095	1.46 [1.22, 1.74]	4.77 [2.77, 6.45]
Lobar pneumonia*	<5 h	1,818	1.50 [1.31, 1.73]	4.58 [3.19, 5.77]
Anemia	<5 h	2,787	1.48 [1.32, 1.66]	4.56 [3.42, 5.58]
Urinary tract infection [UTI]	<5 h	2,221	1.48 [1.30, 1.68]	4.38 [3.13, 5.49]
Allergy to antibiotic agent (excluding penicillin)	<5 h	1,443	1.51 [1.28, 1.77]	4.38 [2.86, 5.67]
Bacterial infections	<5 h	3,029	1.45 [1.30, 1.62]	4.33 [3.22, 5.32]
Acute kidney failure	<5 h	2,686	1.38 [1.23, 1.55]	3.98 [2.71, 5.10]
Type 2 diabetes	<5 h	3,161	1.31 [1.18, 1.45]	3.85 [2.51, 5.05]
Gastrointestinal hemorrhage	<5 h	1,737	1.37 [1.18, 1.58]	3.45 [1.94, 4.76]
Pleural effusion	<5 h	1,875	1.37 [1.19, 1.58]	3.43 [2.01, 4.67]
Constipation	<5 h	3,159	1.36 [1.22, 1.52]	3.31 [2.23, 4.28]
General symptoms and other findings	<5 h	2,781	1.33 [1.18, 1.50]	3.07 [1.91, 4.10]
Major depressive disorder	8–9 h	2,861	1.55 [1.34, 1.78]	2.63 [1.89, 3.27]
Atrial fibrillation and flutter	<5 h	4,880	1.28 [1.17, 1.40]	2.62 [1.73, 3.44]
Osteoarthritis	<5 h	8,752	1.19 [1.11, 1.28]	1.84 [1.16, 2.47]

Models were adjusted for age, sex, TDI, ethnicity, educational attainment, employment status, BMI, and urbanicity, smoking status, alcohol consumption, physical activity, time spent watching TV, time spent using a computer, and mobile phone use duration, residential particulate matter, and nighttime noise levels. HR and 95% CI were calculated to compare the risk of incident disease among individuals with different group of sleep durations, using those with 6–8 hours of sleep as the reference group. * Phenotypes defined exclusively by ICD-10 codes in PhecodeX.

Abbreviations: HR, hazard ratio; CI, confidence interval; PAR, population attributable risk.

### 3.8 Sensitivity analyses

After accounting for total sleep duration, the PheWAS results for sleep stage proportions (REM, light, and deep sleep) revealed distinct patterns compared to those observed for absolute durations. Following Bonferroni correction, an increased REM sleep proportion was significantly associated with a lower risk of 54 disease phenotypes. Conversely, an elevated proportion of light sleep was associated with a higher risk of 28 phenotypes. Regarding deep sleep, an increased proportion was linked to a higher risk of hypoosmolality (hazard ratio 1.17, 95% confidence interval [1.09,1.26]; *p* < 0.001) but a lower risk of major depressive disorder (hazard ratio 0.84, 95% confidence interval [0.80,0.88]; *p* < 0.001) (S1 Fig and S7 Table).

The robustness of our findings was further tested through several rigorous sensitivity analyses. After implementing a 2-year washout period to mitigate reverse causation, 95 out of the original 156 significant associations remained statistically significant (S2 Fig and S8 Table). After adjustment for comorbidity history did not materially alter the observed associations, 131/156 phenotypes remained significant (S3 Fig and S9 Table). The primary PheWAS results remained substantially stable after further adjustment for sleep disorders (151 phenotypes significant) (S4 Fig and S10 Table), seasonality (150 phenotypes significant) (S5 Fig and S11 Table), and total sleep duration for sleep irregular (3 phenotypes significant) (S6 Fig and S12 Table).

## 4. Discussion

Our study provides the most comprehensive map to date of associations between objectively measured, real-world sleep patterns and a broad spectrum of incident diseases in middle-aged and older adults. Leveraging accelerometer-derived sleep patterns in nearly 100,000 UK Biobank participants, we identified over one hundred significant associations between distinct sleep patterns and future health outcomes, offering novel insights into the complexity and clinical relevance of human sleep architecture.

Our findings help clarify the debated role of specific sleep patterns in disease risk. Previous cohort studies, including eHeart and All of Us, have reported that increased REM sleep is linked with lower risk of atrial fibrillation and other cardiovascular diseases [12,47]. Consistent with these observations, we found that greater REM sleep is not only linked to reduced cardiovascular risk but also extends this protective association across 12 major disease categories, including neurological, metabolic, and psychiatric outcomes [48,49]. Additionally, our findings regarding REM and deep sleep as protective factors across multiple neurological, psychiatric, and metabolic outcomes are in line with and broaden earlier studies [48,49], emphasizing the relevance of sleep staging in disease progression. Our analysis also helps resolve prior inconsistencies regarding the effects of light sleep [50–54]. Specifically, increased light sleep as part of prolonged total sleep duration was associated with reduced risk of cardiovascular and metabolic disease, consistent with prior observational studies [50–52]. In contrast, a higher proportion of light sleep, reflecting poorer sleep quality, was associated with elevated risk of mental and musculoskeletal disorders [53,54].

We found that sleep fragmentation, measured through WASO and sleep irregularity, was associated with higher risks of metabolic and psychiatric disorders [53,54], which were consistent with previous studies [53,54]. Our findings highlight that not only the quantity of the sleep is important, sleep regularity and continuity are also critical for optimal health, supporting the recommendations from recent sleep medicine consensus statements [54].

Consistent with previous epidemiological studies, we confirmed a non-linear association between sleep duration and multi-system diseases [36,55,56]. Based on objectively measured sleep duration, our findings suggest that the optimal sleep duration among older adults is 6–8 hours, which is slightly shorter than the commonly suggested range of 7–9 hours. Furthermore, analyses using finer-grained categorization of sleep duration indicated that, the health risks associated with short sleep are predominantly concentrated in cardiometabolic, metabolic, and neurological outcomes, which is consistent with biological pathways involving sympathetic nervous system activation, hypothalamic–pituitary–adrenal axis dysregulation, and increased systemic inflammation [57,58]. By contrast, the associations observed for long sleep duration may reflect a combination of mechanisms, including circadian rhythm disruption [59], and reduced daytime activity [60]. Notably, reverse causation is likely relevant for depressive disorders, as hypersomnia is a well-recognized prodromal or concomitant symptom of depression [61]. In addition, exploratory PAR percentage analyses suggested that a proportion of disease burden may be attributable to sleep deviation from the optimal range. However, given the observational nature of this study, causal interpretations of these associations remain limited, and residual confounding and reverse causation cannot be fully excluded.

This study moves beyond conventional hypothesis-driven analyses of individual sleep traits by providing a comprehensive, population-scale mapping of objective sleep characteristics to a broad spectrum of diseases. This framework enables the identification of distinct patterns of association across multiple health domains, thereby offering a more integrated understanding of how real-world sleep relates to disease risk. Furthermore, these findings provide a systematic foundation for future hypothesis generation and prioritization, helping to guide more targeted mechanistic and interventional studies. However, this study is subject to several limitations. First, as an observational analysis, we cannot exclude the potential for residual confounding despite comprehensive adjustment for known confounders, and therefore causal inferences cannot be made. Second, sleep was assessed over a seven-day period, which may not fully capture habitual sleep patterns or long-term fluctuations. Although repeated assessments with wearable devices have shown moderate to high reproducibility over extended intervals in our study (S13 Table), some degree of misclassification remains possible, which could bias risk estimates toward the null [62]. Third, participant characteristics were recorded on average 5.7 years before sleep measurement; however, prior analyses in this cohort suggest these covariates are relatively stable over time [63]. Fourth, the clinical outcomes in this study were identified solely through inpatient diagnosis records, which did not include data from outpatient clinics, accident and emergency visits, or primary care, potentially leading to an underestimation of the true incidence of certain mild or acute phenotypic outcomes. Fifth, while our study adjusted for general seasonal variations based on the month of accelerometry wear, this approach may not have fully captured the precise daily changes in photoperiod across the British Isles. Consequently, this limitation might lead to an underestimation of the impact of seasonal fluctuations on the associations between sleep patterns and health outcomes. Sixth, reverse causality is a potential concern in our analysis. After implementing a two-year washout period, only approximately 60% of the findings remained statistically significant. This suggests that prodromal or subclinical disease states may have influenced sleep patterns prior to diagnosis, and these findings should be interpreted with caution. Seventh, while the SleepNet algorithm facilitates large-scale sleep staging, it exhibits systematic mean differences compared with gold-standard PSG (such as underestimating REM and overestimating NREM duration). Although these errors are expected to be non-differential and primarily affect absolute values rather than relative risk rankings, these metrics should be interpreted as movement-based estimates rather than exact physiological sleep stages. Finally, the UK Biobank cohort is characterized by a healthy volunteer selection bias, consisting mainly of individuals of White European ancestry with a higher socioeconomic status than the broader population, which may distort the observed health associations and limit the generalizability of our findings [64,65].

In summary, this study maps real-world sleep patterns and their associations with disease risks across various body systems. It clarifies the specific contributions of different sleep stages to disease risk. Moreover, the findings provide additional evidence supporting the role of a 6–8 hours’ sleep duration as a health safeguard for middle-aged and older adults, likely attributable to more favorable distributions of sleep stages. Maintaining a sleep duration of 6–8 hours can effectively reduce the risk of multiple diseases, providing new insights for health promotion and preventive practice.

## Supporting information

S1 Checklist RECORD checklist. Benchimol EI, Smeeth L, Guttmann A, Harron K, Moher D, Petersen I, Sørensen HT, von Elm E, Langan SM, the RECORD Working Committee. The REporting of studies Conducted using Observational Routinely-collected health Data (RECORD) Statement. PLoS Medicine 2015; in press. *Checklist is protected under Creative Commons Attribution (CC BY) license.
(DOCX)

S1 Fig Phenome-wide association studies between sleep stages percentage and incident disease risk. The FDR q significant line is indicated by a blue line, and the *p* value of 0.05 is indicated by a gray line. Filled circles indicate HRs < 1 and *p* < 0.05, empty circles indicate HR > 1 and *p* < 0.05, whereas phenotypes with *p* > 0.05 are indicated by particles under the gray line. *P*-values were calculated using two-sided Wald tests from Cox proportional hazards regression models. **(a)** The accelerometer-derived REM sleep stage; **(b)** the accelerometer-derived deep sleep stage; **(c)** the accelerometer-derived light sleep stage.
(PDF)

S2 Fig Phenome-wide association analyses after a 2-year washout period. The FDR q significant line is indicated by a blue line, and the *p* value of 0.05 is indicated by a gray line. Filled circles indicate HRs < 1 and *p* < 0.05, empty circles indicate HR > 1 and *p* < 0.05, whereas phenotypes with *p* > 0.05 are indicated by particles under the gray line. P-values were calculated using two-sided Wald tests from Cox proportional hazards regression models. **(a)** The accelerometer-derived REM sleep stage; **(b)** the accelerometer-derived light sleep stage; **(c)** the accelerometer-derived deep sleep stage; **(d)** sleep irregularity; **(e)** the accelerometer-derived total sleep duration; **(f)** the accelerometer-derived WASO duration (wakefulness after sleep onset).
(PDF)

S3 Fig Phenome-wide association analyses adjusted for comorbidity history records. The FDR q significant line is indicated by a blue line, and the *p* value of 0.05 is indicated by a gray line. Filled circles indicate HRs < 1 and *p* < 0.05, empty circles indicate HR > 1 and *p* < 0.05, whereas phenotypes with *p* > 0.05 are indicated by particles under the gray line. *P*-values were calculated using two-sided Wald tests from Cox proportional hazards regression models. **(a)** The accelerometer-derived REM sleep stage; **(b)** the accelerometer-derived light sleep stage; **(c)** the accelerometer-derived deep sleep stage; **(d)** sleep irregularity; **(e)** the accelerometer-derived total sleep duration; **(f)** the accelerometer-derived WASO duration (wakefulness after sleep onset).
(PDF)

S4 Fig Phenome-wide association analyses adjusted for sleep disorders history records. The FDR q significant line is indicated by a blue line, and the *p* value of 0.05 is indicated by a gray line. Filled circles indicate HRs < 1 and *p* < 0.05, empty circles indicate HR > 1 and *p* < 0.05, whereas phenotypes with *p* > 0.05 are indicated by particles under the gray line. *P*-values were calculated using two-sided Wald tests from Cox proportional hazards regression models. **(a)** The accelerometer-derived REM sleep stage; **(b)** the accelerometer-derived light sleep stage; **(c)** the accelerometer-derived deep sleep stage; **(d)** sleep irregularity; **(e)** the accelerometer-derived total sleep duration; **(f)** the accelerometer-derived WASO duration (wakefulness after sleep onset).
(PDF)

S5 Fig Phenome-wide association analyses adjusted for seasonality records. The FDR q significant line is indicated by a blue line, and the *p* value of 0.05 is indicated by a gray line. Filled circles indicate HRs < 1 and *p* < 0.05, empty circles indicate HR > 1 and *p* < 0.05, whereas phenotypes with *p* > 0.05 are indicated by particles under the gray line. *P*-values were calculated using two-sided Wald tests from Cox proportional hazards regression models. **(a)** The accelerometer-derived REM sleep stage; **(b)** the accelerometer-derived light sleep stage; **(c)** the accelerometer-derived deep sleep stage; **(d)** sleep irregularity; **(e)** the accelerometer-derived total sleep duration; **(f)** the accelerometer-derived WASO duration (wakefulness after sleep onset).
(PDF)

S6 Fig Phenome-wide association analyses for sleep irregularity adjusted for total sleep duration. The FDR q significant line is indicated by a blue line, and the *p* value of 0.05 is indicated by a gray line. Filled circles indicate HRs < 1 and *p* < 0.05, empty circles indicate HRs > 1 and *p* < 0.05, whereas phenotypes with *p* > 0.05 are indicated by particles under the gray line. *P*-values were calculated using two-sided Wald tests from Cox proportional hazards regression models.
(PDF)

S1 Table UK Biobank study protocol documents. (DOCX)

S2 Table UK Biobank covariates. (DOCX)

S3 Table Associations between sleep patterns and disease phenotypes in main model. Models were adjusted for age, sex, TDI, ethnicity, educational attainment, employment status, BMI, and urbanicity, smoking status, alcohol consumption, physical activity, time spent watching TV, time spent using a computer, and mobile phone use duration, residential particulate matter, and nighttime noise levels. HR, Hazard ratio; CI, confidence intervals; ^a^REM, rapid eye movement; ^b^ WASO, wakefulness after sleep onset duration.
(DOCX)

S4 Table Comparison of significant sleep-disease associations under different multiple testing correction methods. Models were adjusted for age, sex, TDI, ethnicity, educational attainment, employment status, BMI, and urbanicity, smoking status, alcohol consumption, physical activity, time spent watching TV, time spent using a computer, and mobile phone use duration, residential particulate matter, and nighttime noise levels. HR, Hazard ratio; CI, confidence intervals; ^a^REM, rapid eye movement; ^b^WASO, wakefulness after sleep onset duration.
(XLSX)

S5 Table Nonlinear association between total sleep duration and all 1,049 incident diseases. Models were adjusted for age, sex, TDI, ethnicity, educational attainment, employment status, BMI, and urbanicity, smoking status, alcohol consumption, physical activity, time spent watching TV, time spent using a computer, and mobile phone use duration, residential particulate matter, and nighttime noise levels. CI, confidence intervals.
(XLSX)

S6 Table Associations between sleep duration (Three levels) and disease phenotypes. Models were adjusted for age, sex, TDI, ethnicity, educational attainment, employment status, BMI, and urbanicity, smoking status, alcohol consumption, physical activity, time spent watching TV, time spent using a computer, and mobile phone use duration, residential particulate matter, and nighttime noise levels. HR, Hazard ratio; CI, confidence intervals.
(DOCX)

S7 Table Associations between sleep stage percentage and disease phenotypes in main model. Models were adjusted for age, sex, TDI, ethnicity, educational attainment, employment status, BMI, and urbanicity, smoking status, alcohol consumption, physical activity, time spent watching TV, time spent using a computer, and mobile phone use duration, residential particulate matter, and nighttime noise levels. HR, Hazard ratio; CI, confidence intervals; ^a^REM, rapid eye movement.
(DOCX)

S8 Table Associations of sleep patterns with disease phenotypes after a 2-year washout period. Models were adjusted for age, sex, TDI, ethnicity, educational attainment, employment status, BMI, and urbanicity, smoking status, alcohol consumption, physical activity, time spent watching TV, time spent using a computer, and mobile phone use duration, residential particulate matter, and nighttime noise levels. HR, Hazard ratio; CI, confidence intervals; ^a^REM, rapid eye movement; ^b^WASO, wakefulness after sleep onset duration.
(DOCX)

S9 Table Associations between sleep patterns and disease phenotypes with adjustment for comorbidity records. Models were adjusted for above demographic, lifestyle, and environmental exposure covariates, and further adjusted for history comorbidity records. HR, Hazard ratio; CI, confidence intervals; ^a^REM, rapid eye movement; ^b^WASO, wakefulness after sleep onset duration.
(DOCX)

S10 Table Associations between sleep patterns and disease phenotypes with adjustment for sleep disorder records. Models were adjusted for above demographic, lifestyle, and environmental exposure covariates, and further adjusted for history sleep disorder records. HR, Hazard ratio; CI, confidence intervals; ^a^REM, rapid eye movement; ^b^WASO, wakefulness after sleep onset duration.
(DOCX)

S11 Table Associations between sleep patterns and disease phenotypes with adjustment for seasonality records. Models were adjusted for above demographic, lifestyle, and environmental exposure covariates, and further adjusted for season records. HR, Hazard ratio; CI, confidence intervals; ^a^REM, rapid eye movement; ^b^WASO, wakefulness after sleep onset duration.
(DOCX)

S12 Table Associations between sleep irregularity and disease phenotypes with adjustment for total sleep duration. Models were adjusted for age, sex, TDI, ethnicity, educational attainment, employment status, BMI, and urbanicity, smoking status, alcohol consumption, physical activity, time spent watching TV, time spent using a computer, and mobile phone use duration, residential particulate matter, and nighttime noise levels. HR, Hazard ratio; CI, confidence intervals.
(DOCX)

S13 Table Correlations among repeated measures of sleep patterns. A total of 3,167 participants had follow-up repeated measures; T1: 2013 ~ 2015; T2: 2018; ^a^ REM, rapid eye movement; ^b^WASO, wakefulness after sleep onset duration; SD, standard deviation; Pearson correlation tests were conducted to assess the relationships, and corresponding *p*-values were reported.
(DOCX)

## Abbreviations

BMI: body mass index
FDR: false discovery rate
HRs: hazard ratios
IQR: interquartile ranges
LUR: Land Use Regression
PAR%: population attributable risk percent
PheWAS: phenome-wide association study
PSG: polysomnography
RECORD: Reporting of Studies Conducted using Observational Routinely-Collected Data
RCS: restricted cubic spline
REM: rapid eye movement
SD: standard deviation
STROBE: Strengthening the Reporting of Observational Studies in Epidemiology
TDI: Townsend deprivation index
WASO: wake after sleep onset
