# Supplementary material for: Accelerometer-derived real-world sleep stages and risk of incident diseases: A UK Biobank cohort study and phenome-wide association analysis
Source: PLoS Med. 2026 Sep 17;23(9):e1005213. doi: 10.1371/journal.pmed.1005213 (PMC13585268; doi:10.1371/journal.pmed.1005213)
Supplement: Supplementary file 1 [file pmed.1005213.s002.pdf]

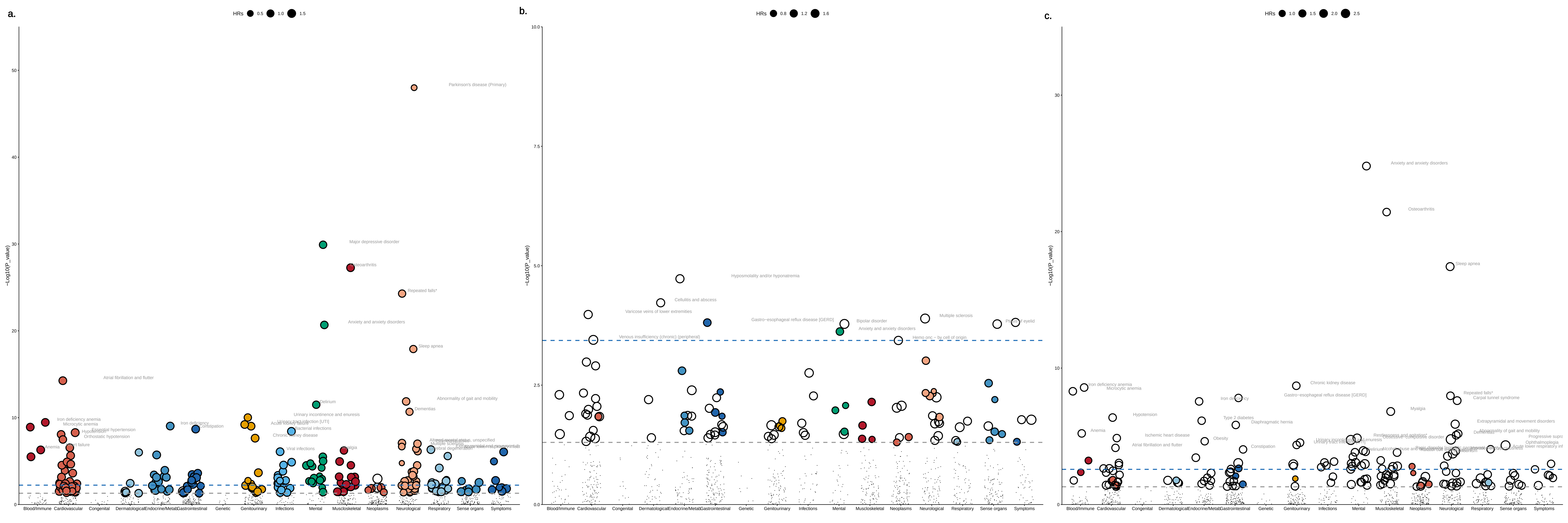

a.
HRs 0.5 1.0 1.5
−Log10(P_value)
Parkinson's disease (Primary)
Major depressive disorder
Osteoarthritis
Repeated falls*
Anxiety and anxiety disorders
Sleep apnea
Atrial fibrillation and flutter
Abnormality of gait and mobility
Delirium
Dementias
Urinary incontinence and enuresis
Iron deficiency anemia
Microcytic anemia
Essential hypertension
Orthostatic hypotension
Iron deficiency
Constipation
Acute kidney failure
Urinary tract infection [UTI]
Chronic kidney disease
Viral infections
Myalgia
Anemia
Blood/Immune
Cardiovascular
Congenital
Dermatological
Endocrine/Metab
Gastrointestinal
Genetic
Genitourinary
Infections
Mental
Muscloskeletal
Neoplasms
Neurological
Respiratory
Sense organs
Symptoms
b.
HRs 0.8 1.2 1.6
−Log10(P_value)
Hyposmolality and/or hyponatremia
Cellulitis and abscess
Varicose veins of lower extremities
Gastro−esophageal reflux disease [GERD]
Bipolar disorder
Anxiety and anxiety disorders
Multiple sclerosis
Ptosis of eyelid
Venous insufficiency (chronic) (peripheral)
Hemo onc − by cell of origin
Blood/Immune
Cardiovascular
Congenital
Dermatological
Endocrine/Metab
Gastrointestinal
Genetic
Genitourinary
Infections
Mental
Muscloskeletal
Neoplasms
Neurological
Respiratory
Sense organs
Symptoms
c.
HRs 1.0 1.5 2.0 2.5
−Log10(P_value)
Anxiety and anxiety disorders
Osteoarthritis
Sleep apnea
Iron deficiency anemia
Microcytic anemia
Chronic kidney disease
Gastro−esophageal reflux disease [GERD]
Repeated falls*
Carpal tunnel syndrome
Iron deficiency
Myalgia
Hypotension
Type 2 diabetes
Diaphragmatic hernia
Extrapyramidal and movement disorders
Anemia
Ischemic heart disease
Obesity
Abnormality of gait and mobility
Atrial fibrillation and flutter
Constipation
Delirium
Blood/Immune
Cardiovascular
Congenital
Dermatological
Endocrine/Metab
Gastrointestinal
Genetic
Genitourinary
Infections
Mental
Muscloskeletal
Neoplasms
Neurological
Respiratory
Sense organs
Symptoms
