# Supplementary material for: Accelerometer-derived real-world sleep stages and risk of incident diseases: A UK Biobank cohort study and phenome-wide association analysis
Source: PLoS Med. 2026 Sep 17;23(9):e1005213. doi: 10.1371/journal.pmed.1005213 (PMC13585268; doi:10.1371/journal.pmed.1005213)
Supplement: Supplementary file 2 [file pmed.1005213.s003.pdf]

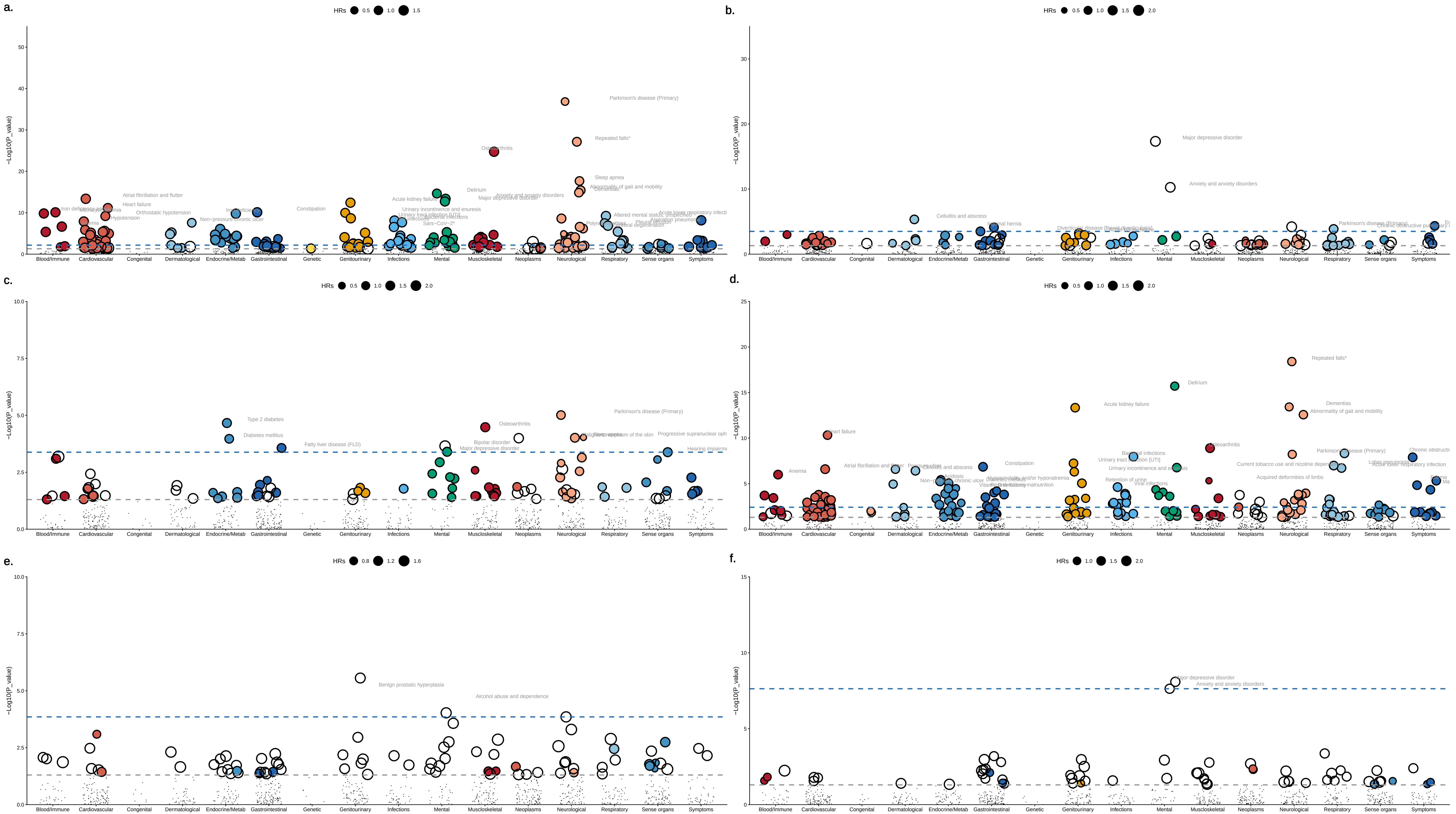

a.
b.
c.
d.
e.
f.
HRs
0.5
1.0
1.5
2.0
0.8
1.2
1.6
−Log10(P_value)
Blood/Immune
Cardiovascular
Congenital
Dermatological
Endocrine/Metab
Gastrointestinal
Genetic
Genitourinary
Infections
Mental
Muscloskeletal
Neoplasms
Neurological
Respiratory
Sense organs
Symptoms
Parkinson's disease (Primary)
Repeated falls*
Osteoarthritis
Sleep apnea
Abnormality of gait and mobility
Dementias
Delirium
Major depressive disorder
Anxiety and anxiety disorders
Atrial fibrillation and flutter
Heart failure
Hypotension
Orthostatic hypotension
Non−pressure chronic ulcer
Constipation
Acute kidney failure
Urinary incontinence and enuresis
Urinary tract infection [UTI]
Sars−CoV−2*
Altered mental status, unspecified
Pleural effusion
Aspiration pneumonia
Cerebral degeneration
Cellulitis and abscess
Inguinal hernia
Diverticular disease [Bowel diverticulosis]
Type 2 diabetes
Diabetes mellitus
Fatty liver disease (FLD)
Bipolar disorder
Progressive supranuclear oph
Hearing impairme
Anemia
Pressure ulcer
Acidosis
Retention of urine
Bacterial infections
Viral infections
Current tobacco use and nicotine dependence
Acquired deformities of limbs
Lobar pneumonia
Acute lower respiratory infection
Chronic obstructiv
Benign prostatic hyperplasia
Alcohol abuse and dependence
