# Supplementary material for: Accelerometer-derived real-world sleep stages and risk of incident diseases: A UK Biobank cohort study and phenome-wide association analysis
Source: PLoS Med. 2026 Sep 17;23(9):e1005213. doi: 10.1371/journal.pmed.1005213 (PMC13585268; doi:10.1371/journal.pmed.1005213)
Supplement: Supplementary file 3 [file pmed.1005213.s004.pdf]

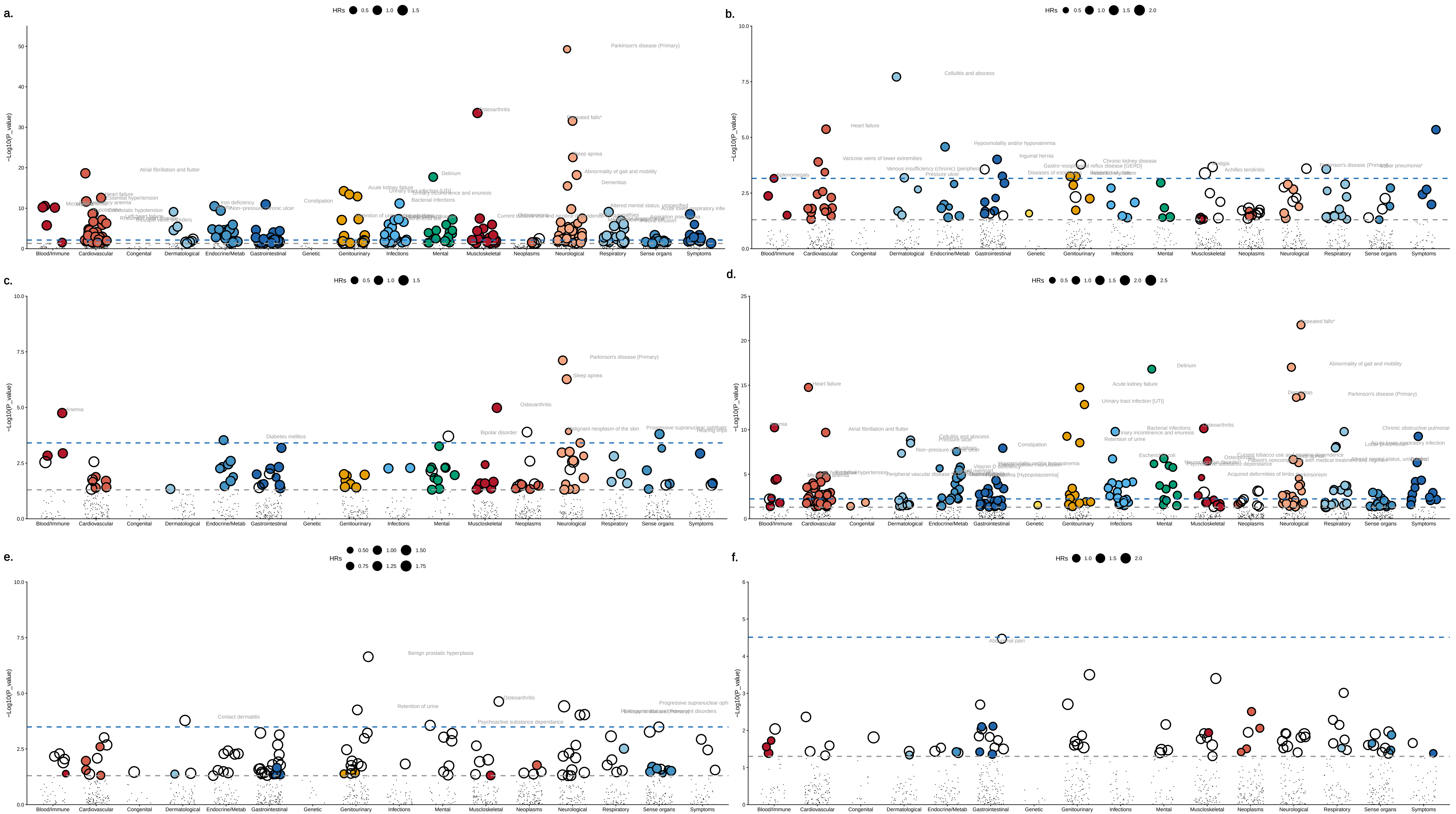

a.
b.
c.
d.
e.
f.
HRs
−Log10(P_value)
Blood/Immune
Cardiovascular
Congenital
Dermatological
Endocrine/Metab
Gastrointestinal
Genetic
Genitourinary
Infections
Mental
Muscloskeletal
Neoplasms
Neurological
Respiratory
Sense organs
Symptoms
Parkinson's disease (Primary)
Osteoarthritis
Repeated falls*
Sleep apnea
Abnormality of gait and mobility
Dementias
Atrial fibrillation and flutter
Delirium
Heart failure
Essential hypertension
Acute kidney failure
Urinary tract infection [UTI]
Urinary incontinence and enuresis
Bacterial infections
Constipation
Iron deficiency
Non−pressure chronic ulcer
Orthostatic hypotension
Altered mental status, unspecified
Aspiration pneumonia
Pleural effusion
Osteoporosis
Cellulitis and abscess
Hyposmolality and/or hyponatremia
Varicose veins of lower extremities
Inguinal hernia
Gastro−esophageal reflux disease [GERD]
Chronic kidney disease
Myalgia
Achilles tendinitis
Lobar pneumonia*
Splenomegaly
Pressure ulcer
Anemia
Diabetes mellitus
Bipolar disorder
Malignant neoplasm of the skin
Chronic obstructive pulmonary
Retention of urine
Escherichia coli
Current tobacco use and nicotine dependence
Psychoactive substance dependance
Acquired deformities of limbs
Parkinsonism
Peripheral vascular disease
Vitamin D deficiency
Benign prostatic hyperplasia
Contact dermatitis
Abdominal pain
