# Supplementary material for: Accelerometer-derived real-world sleep stages and risk of incident diseases: A UK Biobank cohort study and phenome-wide association analysis
Source: PLoS Med. 2026 Sep 17;23(9):e1005213. doi: 10.1371/journal.pmed.1005213 (PMC13585268; doi:10.1371/journal.pmed.1005213)
Supplement: Supplementary file 4 [file pmed.1005213.s005.pdf]

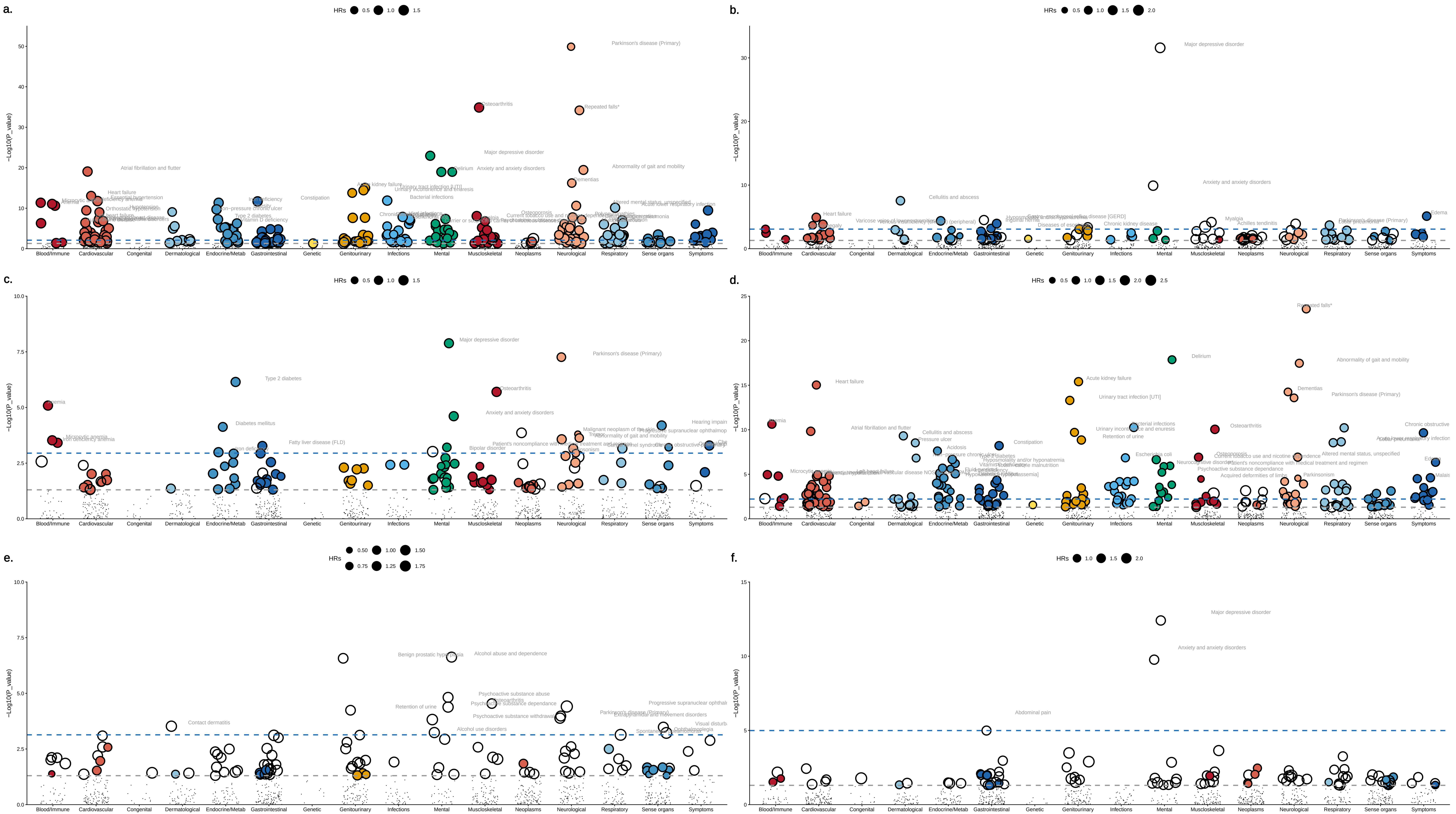

a.
HRs 0.5 1.0 1.5
−Log10(P_value)
Parkinson's disease (Primary)
Osteoarthritis
Repeated falls*
Major depressive disorder
Atrial fibrillation and flutter
Delirium
Anxiety and anxiety disorders
Abnormality of gait and mobility
Dementias
Acute kidney failure
Urinary tract infection [UTI]
Urinary incontinence and enuresis
Bacterial infections
Heart failure
Essential hypertension
Constipation
Iron deficiency
Type 2 diabetes
Vitamin D deficiency
Orthostatic hypotension
Hypotension
Osteoporosis
Altered mental status, unspecified
Acute lower respiratory infection
Blood/Immune
Cardiovascular
Congenital
Dermatological
Endocrine/Metab
Gastrointestinal
Genetic
Genitourinary
Infections
Mental
Muscloskeletal
Neoplasms
Neurological
Respiratory
Sense organs
Symptoms
b.
HRs 0.5 1.0 1.5 2.0
−Log10(P_value)
Major depressive disorder
Anxiety and anxiety disorders
Cellulitis and abscess
Heart failure
Inguinal hernia
Gastro−esophageal reflux disease [GERD]
Diseases of esophagus
Chronic kidney disease
Myalgia
Achilles tendinitis
Parkinson's disease (Primary)
Edema
Blood/Immune
Cardiovascular
Congenital
Dermatological
Endocrine/Metab
Gastrointestinal
Genetic
Genitourinary
Infections
Mental
Muscloskeletal
Neoplasms
Neurological
Respiratory
Sense organs
Symptoms
c.
HRs 0.5 1.0 1.5
−Log10(P_value)
Major depressive disorder
Parkinson's disease (Primary)
Type 2 diabetes
Osteoarthritis
Anxiety and anxiety disorders
Diabetes mellitus
Microcytic anemia
Iron deficiency anemia
Fatty liver disease (FLD)
Bipolar disorder
Progressive supranuclear ophthalmop
Abnormality of gait and mobility
Blood/Immune
Cardiovascular
Congenital
Dermatological
Endocrine/Metab
Gastrointestinal
Genetic
Genitourinary
Infections
Mental
Muscloskeletal
Neoplasms
Neurological
Respiratory
Sense organs
Symptoms
d.
HRs 0.5 1.0 1.5 2.0 2.5
−Log10(P_value)
Repeated falls*
Delirium
Abnormality of gait and mobility
Acute kidney failure
Heart failure
Dementias
Urinary tract infection [UTI]
Parkinson's disease (Primary)
Atrial fibrillation and flutter
Cellulitis and abscess
Pressure ulcer
Bacterial infections
Urinary incontinence and enuresis
Retention of urine
Osteoarthritis
Chronic obstructive
Constipation
Acidosis
Escherichia coli
Altered mental status, unspecified
Hyposmolality and/or hyponatremia
Protein−calorie malnutrition
Neurocognitive disorders
Current tobacco use and nicotine dependence
Patient's noncompliance with medical treatment and regimen
Psychoactive substance dependance
Acquired deformities of limbs
Parkinsonism
Edema
Blood/Immune
Cardiovascular
Congenital
Dermatological
Endocrine/Metab
Gastrointestinal
Genetic
Genitourinary
Infections
Mental
Muscloskeletal
Neoplasms
Neurological
Respiratory
Sense organs
Symptoms
e.
HRs 0.50 1.00 1.50 0.75 1.25 1.75
−Log10(P_value)
Benign prostatic hyperplasia
Alcohol abuse and dependence
Psychoactive substance abuse
Psychoactive substance dependance
Retention of urine
Psychoactive substance withdrawal
Contact dermatitis
Alcohol use disorders
Progressive supranuclear ophthalm
Extrapyramidal and movement disorders
Blood/Immune
Cardiovascular
Congenital
Dermatological
Endocrine/Metab
Gastrointestinal
Genetic
Genitourinary
Infections
Mental
Muscloskeletal
Neoplasms
Neurological
Respiratory
Sense organs
Symptoms
f.
HRs 1.0 1.5 2.0
−Log10(P_value)
Major depressive disorder
Anxiety and anxiety disorders
Abdominal pain
Blood/Immune
Cardiovascular
Congenital
Dermatological
Endocrine/Metab
Gastrointestinal
Genetic
Genitourinary
Infections
Mental
Muscloskeletal
Neoplasms
Neurological
Respiratory
Sense organs
Symptoms
