# Supplementary material for: Accelerometer-derived real-world sleep stages and risk of incident diseases: A UK Biobank cohort study and phenome-wide association analysis
Source: PLoS Med. 2026 Sep 17;23(9):e1005213. doi: 10.1371/journal.pmed.1005213 (PMC13585268; doi:10.1371/journal.pmed.1005213)
Supplement: Supplementary file 5 [file pmed.1005213.s006.pdf]

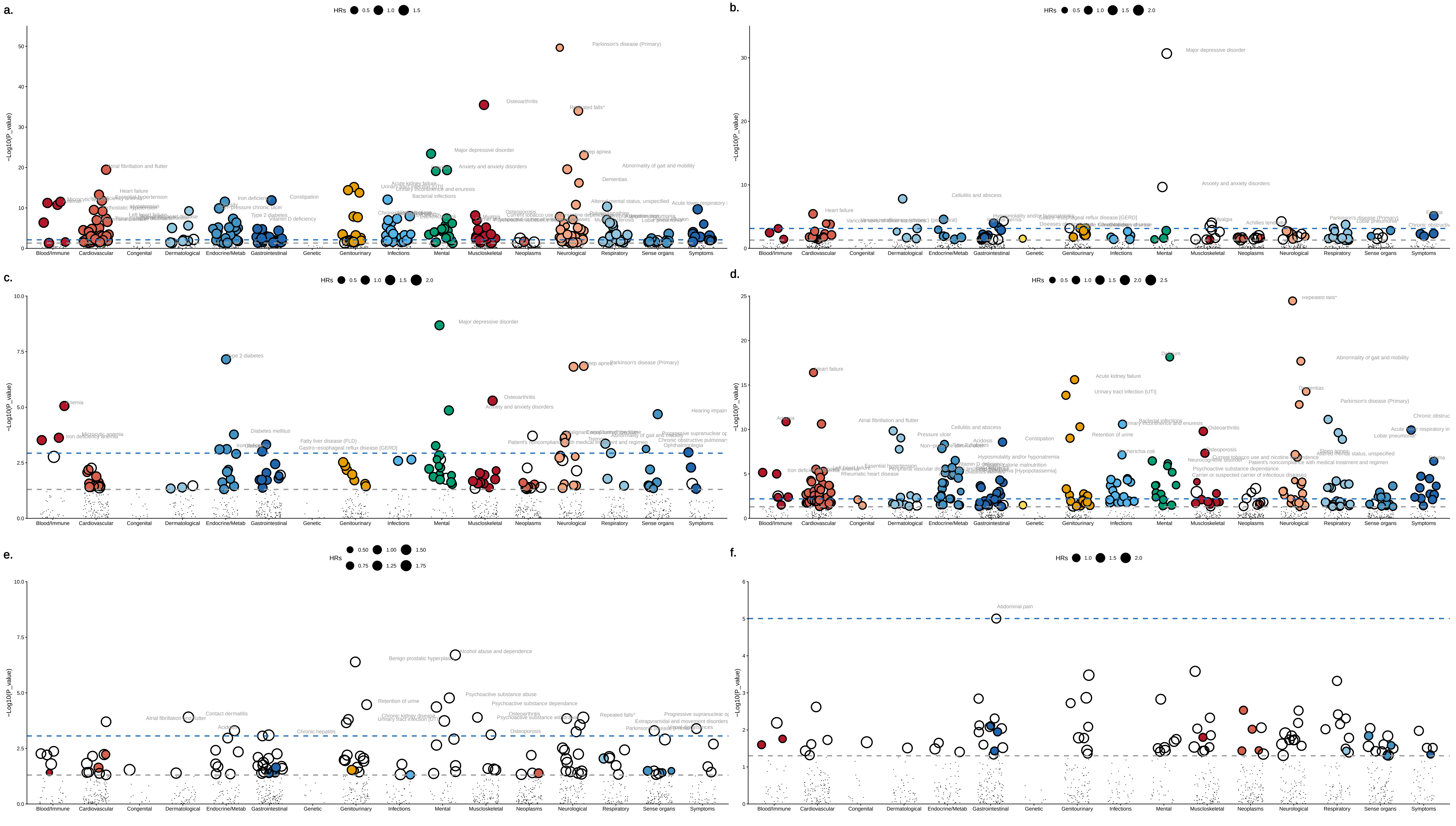
a.
b.
c.
d.
e.
f.
HRs
0.5
1.0
1.5
2.0
2.5
0.50
0.75
1.00
1.25
1.50
1.75
−Log10(P_value)
Blood/Immune
Cardiovascular
Congenital
Dermatological
Endocrine/Metab
Gastrointestinal
Genetic
Genitourinary
Infections
Mental
Muscleskeletal
Neoplasms
Neurological
Respiratory
Sense organs
Symptoms
Parkinson's disease (Primary)
Osteoarthritis
Repeated falls*
Major depressive disorder
Sleep apnea
Anxiety and anxiety disorders
Abnormality of gait and mobility
Atrial fibrillation and flutter
Dementias
Heart failure
Essential hypertension
Iron deficiency
Constipation
Bacterial infections
Urinary incontinence and enuresis
Acute kidney failure
Altered mental status, unspecified
Acute lower respiratory
Type 2 diabetes
Vitamin D deficiency
Orthostatic hypotension
Osteoporosis
Lobar pneumonia*
Cellulitis and abscess
Hyposmolality and/or hyponatremia
Gastro−esophageal reflux disease [GERD]
Myalgia
Achilles tendinitis
Chronic obstructive
Diabetes mellitus
Anemia
Iron deficiency anemia
Microcytic anemia
Fatty liver disease (FLD)
Patient's noncompliance with medical treatment and regimen
Hearing impair
Progressive supranuclear
Chronic obstructive pulmonary
Ophthalmoplegia
Tremor
Delirium
Urinary tract infection [UTI]
Retention of urine
Pressure ulcer
Acidosis
Escherichia coli
Neurocognitive disorder
Current tobacco use and nicotine dependence
Psychoactive substance dependance
Carrier or suspected carrier of infectious diseases
Rheumatic heart disease
Peripheral vascular disease
Sleep apnea
Edema
Alcohol abuse and dependence
Benign prostatic hyperplasia
Psychoactive substance abuse
Psychoactive substance withdrawal
Contact dermatitis
Chronic hepatitis
Chronic kidney disease
Extrapyramidal and movement disorders
Visual disturbances
Abdominal pain
