# Supplementary material for: Accelerometer-derived real-world sleep stages and risk of incident diseases: A UK Biobank cohort study and phenome-wide association analysis
Source: PLoS Med. 2026 Sep 17;23(9):e1005213. doi: 10.1371/journal.pmed.1005213 (PMC13585268; doi:10.1371/journal.pmed.1005213)
Supplement: Supplementary file 6 [file pmed.1005213.s007.pdf]

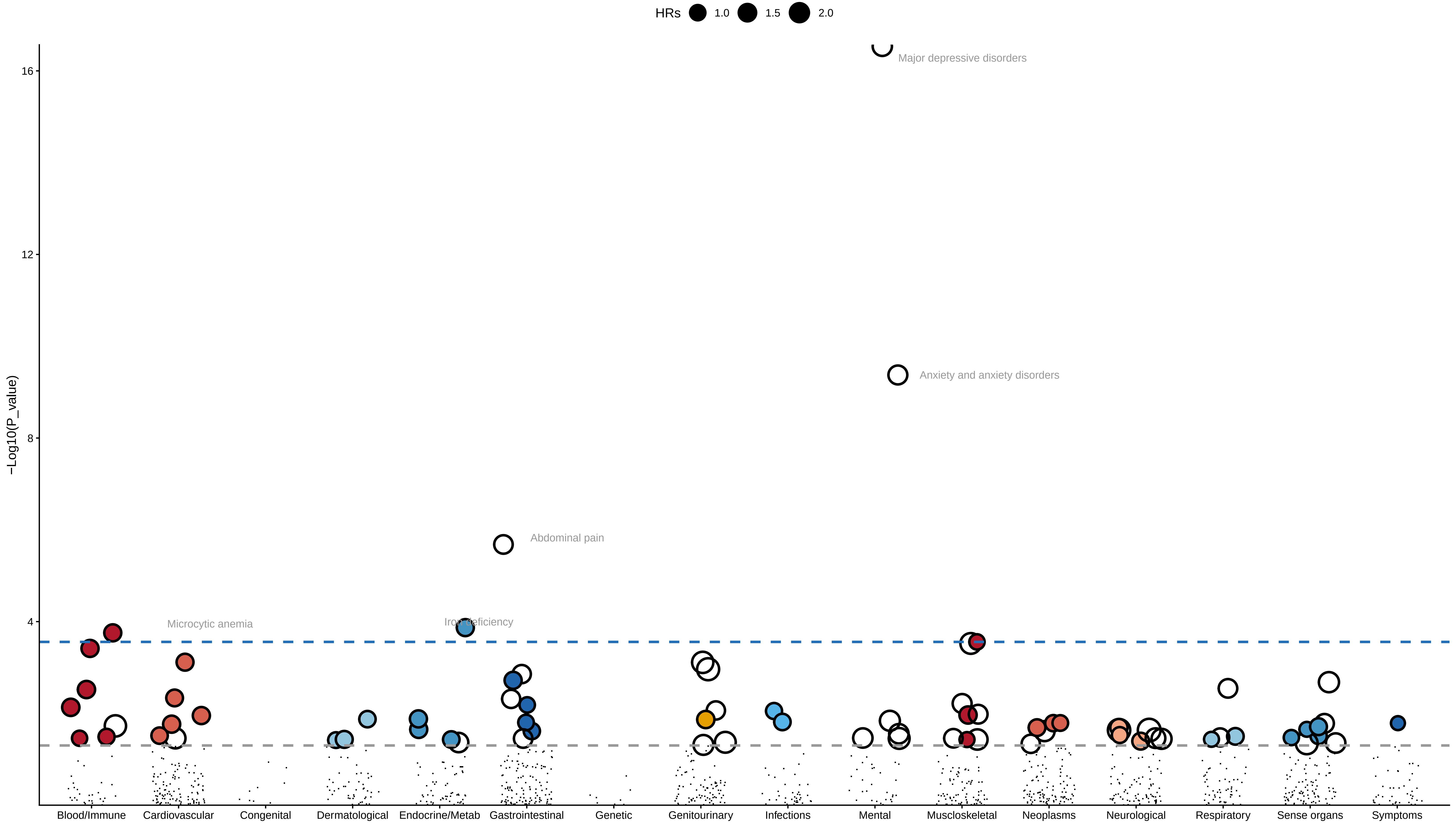

HRs
1.0
1.5
2.0
Major depressive disorders
Anxiety and anxiety disorders
Abdominal pain
Microcytic anemia
Iron deficiency
−Log10(P_value)
16
12
8
4
Blood/Immune
Cardiovascular
Congenital
Dermatological
Endocrine/Metab
Gastrointestinal
Genetic
Genitourinary
Infections
Mental
Muscloskeletal
Neoplasms
Neurological
Respiratory
Sense organs
Symptoms
