# Supplementary material for: Accelerometer-derived real-world sleep stages and risk of incident diseases: A UK Biobank cohort study and phenome-wide association analysis
Source: PLoS Med. 2026 Sep 17;23(9):e1005213. doi: 10.1371/journal.pmed.1005213 (PMC13585268; doi:10.1371/journal.pmed.1005213)
Supplement: Supplementary file 8 [file pmed.1005213.s008.docx]

**S1 Table.** **UK Biobank study protocol documents**

| **Protocol document** | **Link** |
| --- | --- |
| Ethical approval | https://www.ukbiobank.ac.uk/learn-more-about-uk-biobank/about-us/ethics |
| Study participation invitation | https://biobank.ctsu.ox.ac.uk/crystal/refer.cgi?id=100253 |
| First occurrence of health outcomes | https://biobank.ndph.ox.ac.uk/showcase/refer.cgi?id=593 |
| Death registry | https://biobank.ctsu.ox.ac.uk/crystal/refer.cgi?id=115559 |
| Axivity AX3: Participant instructions | https://biobank.ndph.ox.ac.uk/showcase/refer.cgi?id=141141 |
| Axivity AX3: Collection and processing | https://biobank.ndph.ox.ac.uk/showcase/refer.cgi?id=131600 |
| Assessment centre: Reception and consent | https://biobank.ndph.ox.ac.uk/showcase/refer.cgi?id=100230 |
|  | https://biobank.ndph.ox.ac.uk/showcase/ukb/docs/Reception.pdf |
| Assessment centre: Physical measurements | https://biobank.ndph.ox.ac.uk/showcase/refer.cgi?id=100225 |
|  | https://biobank.ndph.ox.ac.uk/showcase/refer.cgi?id=5636 |
|  | https://biobank.ndph.ox.ac.uk/showcase/refer.cgi?id=1227 |
