# Supplementary material for: Accelerometer-derived real-world sleep stages and risk of incident diseases: A UK Biobank cohort study and phenome-wide association analysis
Source: PLoS Med. 2026 Sep 17;23(9):e1005213. doi: 10.1371/journal.pmed.1005213 (PMC13585268; doi:10.1371/journal.pmed.1005213)
Supplement: Supplementary file 9 [file pmed.1005213.s009.docx]

**S2 Table. UK Biobank covariates**

| **Variable (Field-ID)** | **Collection** | **Description** |
| --- | --- | --- |
| Age (21003) | Registry | Participant age at assessment centre visit (years) |
| Sex (31) | Registry | Self-reported sex |
| Townsend deprivation index (189) | Registry | Scores represent deprivation by local area, quantified by average home ownership, car ownership, household overcrowding, and employment rate, and were derived using national census data at time of recruitment. |
| Ethnic background (21000) | Touchscreen Questionnaire | Ethnic group: white and other (mixed, Asian or Asian British, black or black British, Chinese, other) |
| Education (6138) | Touchscreen Questionnaire | Education group: college or university degree and other (A or AS levels, O or GCSEs levels, CSEs, NVQ or HND or HNC, or other professional qualifications) |
| Current employment status (6142) | Touchscreen Questionnaire | Employment group: employed and other (retired, looking after home or family, unable to work because of sickness or disability, unemployed, doing unpaid or voluntary work, full or part-time student) |
| Average total household income before tax (738) | Touchscreen Questionnaire | Income brackets: <£18,000, £18,000-£29,900, £30,000-£51,900, £52,000-£100,000, >£100,000, |
| Urbanicity (20118) | Registry | ‘Urban’ (population ≥ 10,000) and ‘non-urban’ (population < 10,000), defined according to population density of participant’s local area. Derived from the UK Office for National Statistics. |
| Body mass index (21001) | Physical measurements | Weight (kg) / height (m)^2^ |
| Smoking status (20116) | Touchscreen Questionnaire | Smoking status / history: never, previous, current |
| Alcohol intake frequency (1558) | Touchscreen Questionnaire | Daily, 3-4 times per week, 1-2 times per week, 1-3 times per month, special occasions only, never, |
| Physical activity (90012) | Accelerometer | Device average acceleration across one week data collection |
| Length of mobile phone use (1110) | Touchscreen Questionnaire | Never used mobile phone at least once per week, one year or less, two to four years, five to eight years, more than eight years |
| Time spent watching television (1070) | Touchscreen Questionnaire | Time spent watching television per day (hours). |
| Time spent using computer (1080) | Touchscreen Questionnaire | Time spent using computer per day (hours). |
| Particulate matter air pollution (pm2.5); 2010 (24006) | Additional exposures | PM10 (particulate matter with diameter less than or equal to 2.5 micrometres); Land Use Regression (LUR) estimate for annual average 2010. |
| Average night-time sound level of noise pollution (24022) | Additional exposures | Average sound level pressure LAeq overnight 23:00 to 07:00. |
| Start time of wear (90010) | Additional exposures | Seasonality was determined according to the month of accelerometer wear, with seasons classified as follows: March–May (spring), June–August (summer), September–November (autumn), and December–February (winter). |
| Sleep disorders (41270) | Health outcomes | International Classification of Disease version 10 (ICD-10): sleep disorders (F51, F51.2, F51.8, F51.9, G47, G47.8, G47.9); sleep apnoea (G47.3); insomnia (F51.0, G47.0); hypersomnia (F51.1, G47.1); circadian rhythm sleep disorder (G47.2); nightmares (F51.5); sleepwalking (F51.3); narcolepsy (G47.4) |
| Comorbidity diseases (41270) | Health outcomes | International Classification of Disease version 10 (ICD-10): pain (R52.9, F45.4, G50.1, G54.6, R52); chronic pain (R52.1, R52.2, ); major depressive disorders (F32, F32.0, F32.1, F32.2, F32.3, F32.8, F32.9, F33, F33.0, F33.1, F33.2, F33.3, F33.4, F33.8, F33.9); anxiety and anxiety disorders (F93.0, F93.1, F93.2, F40, F41, F41.2, F41.3, F41.8, F41.9, F06.4); alcohol abuse and dependence (F10.1-3); opioid abuse or dependence (F11.1-4); cannabis abuse or dependance (F12.1-4); sedative, hypnotic or anxiolytic-related abuse or dependance (F13.1-4); stimulant abuse or dependance (F14.1-4; F15.1-4); psychoactive substance abuse ( F18.1, F18.3, F18.4, F18.5, F13.1, F15.1, F10.1, F18.6, F18.7, F18.8, F19.0, F12.1, F19.1, F19.3, F19.4, F14.1, F19.5, F19.6, F19.7, F16.1, F11.1, F19.8, F19.9, F18.0); type 2 diabetes (E11, 11.0-9,); hypertension (I67.4, O13, I12, I12.0, I12.9, O10, O16, O10.2, P29.2, O10.9, O11) |
