# Supplementary material for: Accelerometer-derived real-world sleep stages and risk of incident diseases: A UK Biobank cohort study and phenome-wide association analysis
Source: PLoS Med. 2026 Sep 17;23(9):e1005213. doi: 10.1371/journal.pmed.1005213 (PMC13585268; doi:10.1371/journal.pmed.1005213)
Supplement: Supplementary file 11 [file pmed.1005213.s013.docx]

**S6 Table. Associations between** **sleep duration (Three levels) and disease phenotypes**

| **Category** | **Diseases Phenotype** | **Group** | **HR (95% CI)** |
| --- | --- | --- | --- |
| Blood/Immune | Anemia | <6h vs 6-8h | 1.21 (1.12, 1.32) |
| Cardiovascular | Atrial fibrillation and flutter | <6h vs 6-8h | 1.16 (1.09, 1.23) |
| Cardiovascular | Heart failure | <6h vs 6-8h | 1.30 (1.16, 1.45) |
| Dermatological | Cellulitis and abscess | <6h vs 6-8h | 1.30 (1.16, 1.45) |
| Endocrine/Metab | Type 2 diabetes | <6h vs 6-8h | 1.18 (1.09, 1.27) |
| Genitourinary | Acute kidney failure | <6h vs 6-8h | 1.25 (1.16, 1.36) |
| Genitourinary | Retention of urine | <6h vs 6-8h | 1.24 (1.13, 1.36) |
| Genitourinary | Urinary incontinence and enuresis | <6h vs 6-8h | 1.39 (1.20, 1.61) |
| Genitourinary | Urinary tract infection [UTI] | <6h vs 6-8h | 1.30 (1.19, 1.42) |
| Infections | Bacterial infections | <6h vs 6-8h | 1.27 (1.17, 1.37) |
| Infections | Escherichia coli | <6h vs 6-8h | 1.39 (1.20, 1.62) |
| Mental | Delirium | <6h vs 6-8h | 1.52 (1.33, 1.75) |
| Mental | Major depressive disorder | >8h vs 6-8h | 1.60 (1.39, 1.84) |
| Muscloskeletal | Osteoarthritis | <6h vs 6-8h | 1.11 (1.06, 1.16) |
| Neurological | Abnormality of gait and mobility | <6h vs 6-8h | 1.48 (1.32, 1.65) |
| Neurological | Dementias | <6h vs 6-8h | 1.76 (1.45, 2.14) |
| Neurological | Parkinson's disease (Primary) | <6h vs 6-8h | 1.68 (1.37, 2.05) |
| Neurological | Repeated falls* | <6h vs 6-8h | 1.49 (1.34, 1.65) |
| Neurological | Sleep apnea | <6h vs 6-8h | 1.41 (1.23, 1.62) |
| Respiratory | Acute lower respiratory infection | <6h vs 6-8h | 1.26 (1.14, 1.39) |
| Respiratory | Chronic obstructive pulmonary disease [COPD] | <6h vs 6-8h | 1.24 (1.13, 1.37) |
| Respiratory | Lobar pneumonia* | <6h vs 6-8h | 1.34 (1.21, 1.48) |
| Symptoms | Allergy to antibiotic agent (excluding penicillin) | <6h vs 6-8h | 1.27 (1.13, 1.42) |
| Symptoms | General symptoms and other findings | <6h vs 6-8h | 1.21 (1.12, 1.31) |
