# Supplementary material for: Accelerometer-derived real-world sleep stages and risk of incident diseases: A UK Biobank cohort study and phenome-wide association analysis
Source: PLoS Med. 2026 Sep 17;23(9):e1005213. doi: 10.1371/journal.pmed.1005213 (PMC13585268; doi:10.1371/journal.pmed.1005213)
Supplement: Supplementary file 12 [file pmed.1005213.s014.docx]

**S7 Table. Associations between** **sleep stage percentage and disease phenotypes in main model**

| **Category** | **Diseases Phenotype** | **Sleep Pattern** | **HR [95% CI]** |
| --- | --- | --- | --- |
| Blood/Immune | Anemia | REM proportion | 0.87 [0.83, 0.92] |
| Blood/Immune | Autoimmune disease | REM proportion | 0.89 [0.84, 0.93] |
| Blood/Immune | Iron deficiency anemia | REM proportion | 0.83 [0.78, 0.88] |
| Blood/Immune | Microcytic anemia | REM proportion | 0.84 [0.79, 0.89] |
| Cardiovascular | Atrial fibrillation and flutter | REM proportion | 0.85 [0.82, 0.89] |
| Cardiovascular | Complications and ill-defined descriptions of heart disease | REM proportion | 0.84 [0.77, 0.91] |
| Cardiovascular | Essential hypertension | REM proportion | 0.93 [0.90, 0.95] |
| Cardiovascular | Heart failure | REM proportion | 0.82 [0.76, 0.88] |
| Cardiovascular | Hypotension | REM proportion | 0.81 [0.76, 0.87] |
| Cardiovascular | Ischemic heart disease | REM proportion | 0.88 [0.84, 0.93] |
| Cardiovascular | Left heart failure | REM proportion | 0.81 [0.74, 0.90] |
| Cardiovascular | Orthostatic hypotension | REM proportion | 0.75 [0.67, 0.83] |
| Cardiovascular | Rheumatic heart disease | REM proportion | 0.84 [0.78, 0.91] |
| Cardiovascular | Tricuspid valve disorders | REM proportion | 0.81 [0.74, 0.89] |
| Dermatological | Non-pressure chronic ulcer | REM proportion | 0.66 [0.56, 0.78] |
| Endocrine/Metab | Iron deficiency | REM proportion | 0.84 [0.79, 0.89] |
| Endocrine/Metab | Obesity | REM proportion | 0.90 [0.86, 0.94] |
| Gastrointestinal | Constipation | REM proportion | 0.86 [0.81, 0.90] |
| Genitourinary | Acute kidney failure | REM proportion | 0.84 [0.80, 0.89] |
| Genitourinary | Chronic kidney disease | REM proportion | 0.86 [0.81, 0.91] |
| Genitourinary | Urinary incontinence and enuresis | REM proportion | 0.71 [0.64, 0.79] |
| Genitourinary | Urinary tract infection [UTI] | REM proportion | 0.83 [0.78, 0.88] |
| Infections | Bacterial infections | REM proportion | 0.86 [0.81, 0.90] |
| Infections | Escherichia coli | REM proportion | 0.80 [0.72, 0.89] |
| Infections | Sars-CoV-2* | REM proportion | 0.83 [0.76, 0.90] |
| Infections | Viral infections | REM proportion | 0.86 [0.81, 0.91] |
| Mental | Alcohol abuse and dependence | REM proportion | 0.77 [0.69, 0.86] |
| Mental | Anxiety and anxiety disorders | REM proportion | 0.76 [0.72, 0.81] |
| Mental | Current tobacco use and nicotine dependence | REM proportion | 0.86 [0.80, 0.92] |
| Mental | Delirium | REM proportion | 0.71 [0.64, 0.78] |
| Mental | Major depressive disorder | REM proportion | 0.73 [0.69, 0.77] |
| Mental | Psychoactive substance abuse | REM proportion | 0.76 [0.68, 0.86] |
| Mental | Psychoactive substance dependance | REM proportion | 0.63 [0.50, 0.78] |
| Mental | Restlessness and agitation* | REM proportion | 0.54 [0.41, 0.71] |
| Muscloskeletal | Bursitis | REM proportion | 0.73 [0.63, 0.85] |
| Muscloskeletal | Myalgia | REM proportion | 0.71 [0.62, 0.81] |
| Muscloskeletal | Osteoarthritis | REM proportion | 0.84 [0.82, 0.87] |
| Muscloskeletal | Osteoporosis | REM proportion | 0.87 [0.81, 0.92] |
| Neurological | Abnormality of gait and mobility | REM proportion | 0.75 [0.70, 0.81] |
| Neurological | Altered mental status, unspecified | REM proportion | 0.75 [0.68, 0.83] |
| Neurological | Alzheimer's disease | REM proportion | 0.71 [0.60, 0.83] |
| Neurological | Cerebral degeneration | REM proportion | 0.63 [0.53, 0.76] |
| Neurological | Dementias | REM proportion | 0.62 [0.54, 0.72] |
| Neurological | Extrapyramidal and movement disorders | REM proportion | 0.54 [0.43, 0.69] |
| Neurological | Multiple sclerosis | REM proportion | 0.47 [0.36, 0.63] |
| Neurological | Parkinson's disease (Primary) | REM proportion | 0.31 [0.26, 0.36] |
| Neurological | Parkinsonism | REM proportion | 0.27 [0.15, 0.49] |
| Neurological | Polyneuropathies | REM proportion | 0.74 [0.66, 0.82] |
| Neurological | Repeated falls* | REM proportion | 0.68 [0.63, 0.73] |
| Neurological | Sleep apnea | REM proportion | 0.65 [0.59, 0.71] |
| Respiratory | Acute lower respiratory infection | REM proportion | 0.84 [0.79, 0.90] |
| Respiratory | Aspiration pneumonia | REM proportion | 0.69 [0.59, 0.80] |
| Symptoms | General symptoms and other findings | REM proportion | 0.87 [0.83, 0.92] |
| Symptoms | Wheelchair dependence | REM proportion | 0.57 [0.44, 0.73] |
| Endocrine/Metab | Hyposmolality and/or hyponatremia | Deep proportion | 1.17 [1.09, 1.26] |
| Mental | Major depressive disorder | Deep proportion | 0.84 [0.80, 0.88] |
| Blood/Immune | Anemia | Light proportion | 1.11 [1.06, 1.16] |
| Blood/Immune | Iron deficiency anemia | Light proportion | 1.16 [1.11, 1.22] |
| Blood/Immune | Microcytic anemia | Light proportion | 1.16 [1.10, 1.22] |
| Cardiovascular | Hypotension | Light proportion | 1.17 [1.10, 1.24] |
| Cardiovascular | Ischemic heart disease | Light proportion | 1.10 [1.05, 1.15] |
| Endocrine/Metab | Iron deficiency | Light proportion | 1.15 [1.09, 1.20] |
| Endocrine/Metab | Obesity | Light proportion | 1.08 [1.04, 1.12] |
| Endocrine/Metab | Type 2 diabetes | Light proportion | 1.11 [1.07, 1.16] |
| Gastrointestinal | Diaphragmatic hernia | Light proportion | 1.10 [1.06, 1.14] |
| Gastrointestinal | Gastro-esophageal reflux disease [GERD] | Light proportion | 1.10 [1.06, 1.13] |
| Genitourinary | Chronic kidney disease | Light proportion | 1.15 [1.10, 1.20] |
| Genitourinary | Urinary incontinence and enuresis | Light proportion | 1.19 [1.10, 1.30] |
| Genitourinary | Urinary tract infection [UTI] | Light proportion | 1.11 [1.06, 1.17] |
| Mental | Anxiety and anxiety disorders | Light proportion | 1.28 [1.23, 1.35] |
| Mental | Major depressive disorder | Light proportion | 1.40 [1.34, 1.46] |
| Mental | Obsessive-compulsive disorder | Light proportion | 2.27 [1.56, 3.31] |
| Mental | Restlessness and agitation* | Light proportion | 1.60 [1.29, 1.97] |
| Muscloskeletal | Myalgia | Light proportion | 1.34 [1.20, 1.49] |
| Muscloskeletal | Osteoarthritis | Light proportion | 1.14 [1.11, 1.17] |
| Neurological | Abnormality of gait and mobility | Light proportion | 1.16 [1.09, 1.23] |
| Neurological | Carpal tunnel syndrome | Light proportion | 1.27 [1.17, 1.38] |
| Neurological | Dementias | Light proportion | 1.27 [1.15, 1.42] |
| Neurological | Extrapyramidal and movement disorders | Light proportion | 1.55 [1.30, 1.85] |
| Neurological | Parkinson's disease (Primary) | Light proportion | 1.96 [1.77, 2.17] |
| Neurological | Progressive supranuclear ophthalmoplegia [Steele-Richardson-Olszewski]* | Light proportion | 2.51 [1.65, 3.81] |
| Neurological | Repeated falls* | Light proportion | 1.18 [1.12, 1.26] |
| Neurological | Sleep apnea | Light proportion | 1.39 [1.29, 1.50] |
| Sense organs | Ophthalmoplegia | Light proportion | 2.16 [1.49, 3.12] |
