# Supplementary material for: Accelerometer-derived real-world sleep stages and risk of incident diseases: A UK Biobank cohort study and phenome-wide association analysis
Source: PLoS Med. 2026 Sep 17;23(9):e1005213. doi: 10.1371/journal.pmed.1005213 (PMC13585268; doi:10.1371/journal.pmed.1005213)
Supplement: Supplementary file 15 [file pmed.1005213.s017.docx]

**S10 Table. Associations between sleep patterns and disease phenotypes with adjustment for sleep disorder records**

| **Category** | **Diseases Phenotype** | **Sleep Pattern** | **HR [95% CI] with adjustment** | **HR [95% CI] without adjustment** |
| --- | --- | --- | --- | --- |
| Blood/Immune | Anemia | REM sleep | 0.83 [0.79, 0.88] | 0.83 [0.79, 0.88] |
| Blood/Immune | Autoimmune disease | REM sleep | 0.88 [0.84, 0.93] | 0.88 [0.84, 0.92] |
| Blood/Immune | Iron deficiency anemia | REM sleep | 0.81 [0.76, 0.86] | 0.81 [0.76, 0.86] |
| Blood/Immune | Microcytic anemia | REM sleep | 0.82 [0.77, 0.86] | 0.81 [0.77, 0.86] |
| Cardiovascular | Atrial fibrillation and flutter | REM sleep | 0.83 [0.79, 0.86] | 0.83 [0.79, 0.86] |
| Cardiovascular | Cardiomegaly | REM sleep | 0.84 [0.78, 0.91] | 0.84 [0.77, 0.91] |
| Cardiovascular | Complications and ill-defined descriptions of heart disease | REM sleep | 0.83 [0.76, 0.90] | 0.83 [0.76, 0.90] |
| Cardiovascular | Essential hypertension | REM sleep | 0.91 [0.89, 0.93] | 0.91 [0.89, 0.93] |
| Cardiovascular | Heart failure | REM sleep | 0.74 [0.68, 0.80] | 0.74 [0.68, 0.80] |
| Cardiovascular | Hypotension | REM sleep | 0.79 [0.74, 0.85] | 0.79 [0.74, 0.85] |
| Cardiovascular | Ischemic heart disease | REM sleep | 0.87 [0.83, 0.92] | 0.87 [0.83, 0.92] |
| Cardiovascular | Left heart failure | REM sleep | 0.76 [0.69, 0.84] | 0.76 [0.69, 0.84] |
| Cardiovascular | Mitral valve disorders | REM sleep | 0.83 [0.76, 0.90] | 0.82 [0.75, 0.90] |
| Cardiovascular | Orthostatic hypotension | REM sleep | 0.72 [0.65, 0.80] | 0.72 [0.65, 0.80] |
| Cardiovascular | Rheumatic heart disease | REM sleep | 0.81 [0.75, 0.88] | 0.81 [0.75, 0.88] |
| Cardiovascular | Tricuspid valve disorders | REM sleep | 0.78 [0.70, 0.86] | 0.77 [0.70, 0.85] |
| Dermatological | Cellulitis and abscess | REM sleep | 0.84 [0.78, 0.91] | 0.84 [0.78, 0.91] |
| Dermatological | Non-pressure chronic ulcer | REM sleep | 0.58 [0.48, 0.69] | 0.57 [0.48, 0.68] |
| Dermatological | Pressure ulcer | REM sleep | 0.73 [0.64, 0.83] | 0.73 [0.64, 0.83] |
| Endocrine/Metab | Acidosis | REM sleep | 0.73 [0.63, 0.84] | 0.73 [0.63, 0.84] |
| Endocrine/Metab | Disorders of calcium metabolism | REM sleep | 0.76 [0.67, 0.86] | 0.76 [0.67, 0.87] |
| Endocrine/Metab | Fluid overload | REM sleep | 0.71 [0.61, 0.82] | 0.71 [0.61, 0.82] |
| Endocrine/Metab | Hyperosmolality and/or hypernatremia | REM sleep | 0.60 [0.47, 0.75] | 0.60 [0.47, 0.75] |
| Endocrine/Metab | Hypokalemia [Hypopotassemia] | REM sleep | 0.80 [0.73, 0.88] | 0.80 [0.73, 0.88] |
| Endocrine/Metab | Hyposmolality and/or hyponatremia | REM sleep | 0.83 [0.77, 0.90] | 0.83 [0.77, 0.90] |
| Endocrine/Metab | Iron deficiency | REM sleep | 0.82 [0.77, 0.86] | 0.82 [0.77, 0.86] |
| Endocrine/Metab | Obesity | REM sleep | 0.86 [0.83, 0.90] | 0.86 [0.82, 0.90] |
| Endocrine/Metab | Type 2 diabetes | REM sleep | 0.87 [0.83, 0.91] | 0.87 [0.82, 0.91] |
| Endocrine/Metab | Vitamin D deficiency | REM sleep | 0.77 [0.69, 0.85] | 0.76 [0.69, 0.85] |
| Gastrointestinal | Constipation | REM sleep | 0.83 [0.79, 0.88] | 0.83 [0.79, 0.87] |
| Gastrointestinal | Esophageal ulcer | REM sleep | 0.71 [0.60, 0.83] | 0.71 [0.61, 0.83] |
| Gastrointestinal | Fecal incontinence and encopresis | REM sleep | 0.74 [0.64, 0.85] | 0.74 [0.64, 0.85] |
| Genitourinary | Acute kidney failure | REM sleep | 0.80 [0.75, 0.84] | 0.79 [0.75, 0.84] |
| Genitourinary | Chronic kidney disease | REM sleep | 0.86 [0.81, 0.91] | 0.86 [0.81, 0.90] |
| Genitourinary | Retention of urine | REM sleep | 0.84 [0.79, 0.89] | 0.84 [0.79, 0.89] |
| Genitourinary | Urinary incontinence and enuresis | REM sleep | 0.66 [0.59, 0.73] | 0.66 [0.59, 0.73] |
| Genitourinary | Urinary tract infection [UTI] | REM sleep | 0.78 [0.73, 0.83] | 0.78 [0.73, 0.83] |
| Infections | Bacterial infections | REM sleep | 0.83 [0.79, 0.87] | 0.83 [0.78, 0.87] |
| Infections | Carrier or suspected carrier of infectious diseases | REM sleep | 0.65 [0.54, 0.77] | 0.64 [0.54, 0.76] |
| Infections | Drug resistant microorganisms | REM sleep | 0.66 [0.55, 0.79] | 0.66 [0.55, 0.78] |
| Infections | Escherichia coli | REM sleep | 0.75 [0.67, 0.83] | 0.75 [0.67, 0.83] |
| Infections | Sars-CoV-2* | REM sleep | 0.78 [0.72, 0.86] | 0.78 [0.72, 0.86] |
| Infections | Sepsis | REM sleep | 0.87 [0.81, 0.93] | 0.87 [0.81, 0.93] |
| Infections | Viral infections | REM sleep | 0.84 [0.78, 0.89] | 0.83 [0.78, 0.89] |
| Mental | Alcohol abuse and dependence | REM sleep | 0.76 [0.68, 0.86] | 0.76 [0.68, 0.85] |
| Mental | Anxiety and anxiety disorders | REM sleep | 0.77 [0.73, 0.82] | 0.77 [0.73, 0.81] |
| Mental | Bipolar disorder | REM sleep | 0.54 [0.40, 0.72] | NA |
| Mental | Cannabis abuse or dependance | REM sleep | 0.26 [0.14, 0.50] | 0.26 [0.14, 0.49] |
| Mental | Current tobacco use and nicotine dependence | REM sleep | 0.81 [0.76, 0.88] | 0.81 [0.76, 0.88] |
| Mental | Delirium | REM sleep | 0.63 [0.57, 0.69] | 0.62 [0.56, 0.69] |
| Mental | Major depressive disorder | REM sleep | 0.76 [0.72, 0.80] | 0.75 [0.71, 0.80] |
| Mental | Neurocognitive disorder* | REM sleep | 0.56 [0.43, 0.74] | 0.56 [0.43, 0.74] |
| Mental | Psychoactive substance abuse | REM sleep | 0.77 [0.68, 0.87] | 0.76 [0.68, 0.86] |
| Mental | Psychoactive substance dependance | REM sleep | 0.55 [0.44, 0.70] | 0.55 [0.44, 0.70] |
| Mental | Restlessness and agitation* | REM sleep | 0.52 [0.39, 0.70] | 0.52 [0.39, 0.70] |
| Muscloskeletal | Bursitis | REM sleep | 0.71 [0.61, 0.83] | 0.71 [0.61, 0.83] |
| Muscloskeletal | Myalgia | REM sleep | 0.70 [0.61, 0.80] | 0.69 [0.61, 0.79] |
| Muscloskeletal | Osteoarthritis | REM sleep | 0.82 [0.80, 0.85] | 0.82 [0.79, 0.85] |
| Muscloskeletal | Osteoporosis | REM sleep | 0.83 [0.78, 0.89] | 0.83 [0.78, 0.88] |
| Muscloskeletal | Rotator cuff tear or rupture | REM sleep | 0.75 [0.66, 0.86] | 0.75 [0.66, 0.86] |
| Neurological | Abnormality of gait and mobility | REM sleep | 0.68 [0.63, 0.74] | 0.68 [0.63, 0.74] |
| Neurological | Altered mental status, unspecified | REM sleep | 0.69 [0.62, 0.77] | 0.69 [0.62, 0.77] |
| Neurological | Alzheimer's disease | REM sleep | 0.69 [0.59, 0.81] | 0.69 [0.59, 0.81] |
| Neurological | Carpal tunnel syndrome | REM sleep | 0.80 [0.73, 0.89] | 0.80 [0.73, 0.89] |
| Neurological | Cerebral degeneration | REM sleep | 0.60 [0.50, 0.72] | 0.60 [0.50, 0.72] |
| Neurological | Dementias | REM sleep | 0.54 [0.47, 0.62] | 0.54 [0.47, 0.62] |
| Neurological | Extrapyramidal and movement disorders | REM sleep | 0.55 [0.43, 0.70] | 0.55 [0.43, 0.70] |
| Neurological | Multiple sclerosis | REM sleep | 0.48 [0.36, 0.64] | 0.48 [0.36, 0.64] |
| Neurological | Parkinson's disease (Primary) | REM sleep | 0.28 [0.24, 0.33] | 0.28 [0.24, 0.33] |
| Neurological | Parkinsonism | REM sleep | 0.20 [0.11, 0.39] | 0.20 [0.11, 0.39] |
| Neurological | Polyneuropathies | REM sleep | 0.72 [0.64, 0.81] | 0.72 [0.64, 0.80] |
| Neurological | Repeated falls* | REM sleep | 0.62 [0.57, 0.67] | 0.62 [0.57, 0.67] |
| Neurological | Speech disturbance | REM sleep | 0.64 [0.52, 0.79] | 0.63 [0.51, 0.78] |
| Neurological | Symptoms and signs involving cognitive functions and awareness | REM sleep | 0.68 [0.57, 0.80] | 0.68 [0.57, 0.80] |
| Respiratory | Acute lower respiratory infection | REM sleep | 0.80 [0.74, 0.85] | 0.79 [0.74, 0.85] |
| Respiratory | Aspiration pneumonia | REM sleep | 0.64 [0.55, 0.76] | 0.64 [0.55, 0.76] |
| Respiratory | Chronic obstructive pulmonary disease [COPD] | REM sleep | 0.86 [0.81, 0.92] | 0.86 [0.80, 0.91] |
| Respiratory | Lobar pneumonia* | REM sleep | 0.85 [0.79, 0.90] | 0.85 [0.79, 0.90] |
| Respiratory | Pleural effusion | REM sleep | 0.85 [0.79, 0.90] | 0.84 [0.79, 0.90] |
| Symptoms | General symptoms and other findings | REM sleep | 0.84 [0.80, 0.89] | 0.84 [0.79, 0.89] |
| Symptoms | Wheelchair dependence | REM sleep | 0.51 [0.39, 0.67] | 0.51 [0.39, 0.67] |
| Blood/Immune | Anemia | Deep sleep | 0.89 [0.85, 0.94] | 0.89 [0.85, 0.94] |
| Endocrine/Metab | Type 2 diabetes | Deep sleep | 0.89 [0.85, 0.93] | 0.89 [0.85, 0.93] |
| Mental | Anxiety and anxiety disorders | Deep sleep | 0.89 [0.85, 0.94] | 0.90 [0.85, 0.94] |
| Mental | Major depressive disorder | Deep sleep | 0.86 [0.82, 0.91] | 0.86 [0.82, 0.91] |
| Muscloskeletal | Osteoarthritis | Deep sleep | 0.93 [0.91, 0.96] | 0.93 [0.91, 0.96] |
| Neurological | Parkinson's disease (Primary) | Deep sleep | 0.70 [0.62, 0.80] | 0.70 [0.62, 0.80] |
| Cardiovascular | Heart failure | Light sleep | 0.87 [0.81, 0.92] | 0.86 [0.81, 0.92] |
| Dermatological | Cellulitis and abscess | Light sleep | 0.83 [0.78, 0.89] | 0.83 [0.78, 0.89] |
| Endocrine/Metab | Hyposmolality and/or hyponatremia | Light sleep | 0.86 [0.81, 0.93] | 0.86 [0.81, 0.93] |
| Gastrointestinal | Gastro-esophageal reflux disease [GERD] | Light sleep | 1.07 [1.04, 1.10] | 1.07 [1.03, 1.10] |
| Mental | Anxiety and anxiety disorders | Light sleep | 1.16 [1.11, 1.22] | 1.16 [1.11, 1.22] |
| Mental | Major depressive disorder | Light sleep | 1.31 [1.25, 1.37] | 1.31 [1.25, 1.36] |
| Symptoms | Edema | Light sleep | 0.83 [0.77, 0.90] | 0.83 [0.77, 0.90] |
| Blood/Immune | Anemia | Sleep duration | 0.86 [0.82, 0.90] | 0.86 [0.82, 0.90] |
| Blood/Immune | Iron deficiency anemia | Sleep duration | 0.90 [0.85, 0.94] | 0.90 [0.85, 0.94] |
| Blood/Immune | Microcytic anemia | Sleep duration | 0.90 [0.85, 0.94] | 0.90 [0.85, 0.94] |
| Cardiovascular | Atrial fibrillation and flutter | Sleep duration | 0.89 [0.86, 0.92] | 0.89 [0.86, 0.92] |
| Cardiovascular | Essential hypertension | Sleep duration | 0.95 [0.93, 0.97] | 0.95 [0.93, 0.97] |
| Cardiovascular | Heart failure | Sleep duration | 0.78 [0.73, 0.83] | 0.78 [0.73, 0.82] |
| Cardiovascular | Left heart failure | Sleep duration | 0.84 [0.77, 0.91] | 0.84 [0.77, 0.91] |
| Cardiovascular | Peripheral vascular disease NOS [includes PAD] | Sleep duration | 0.80 [0.72, 0.88] | 0.79 [0.72, 0.88] |
| Dermatological | Cellulitis and abscess | Sleep duration | 0.82 [0.77, 0.87] | 0.82 [0.77, 0.87] |
| Dermatological | Non-pressure chronic ulcer | Sleep duration | 0.71 [0.63, 0.81] | 0.71 [0.62, 0.80] |
| Dermatological | Pressure ulcer | Sleep duration | 0.74 [0.67, 0.82] | 0.74 [0.67, 0.82] |
| Endocrine/Metab | Acidosis | Sleep duration | 0.74 [0.67, 0.82] | 0.74 [0.66, 0.82] |
| Endocrine/Metab | Diabetes mellitus | Sleep duration | 0.80 [0.72, 0.89] | 0.80 [0.72, 0.89] |
| Endocrine/Metab | Fluid overload | Sleep duration | 0.77 [0.69, 0.86] | 0.77 [0.69, 0.87] |
| Endocrine/Metab | Hypokalemia [Hypopotassemia] | Sleep duration | 0.84 [0.78, 0.91] | 0.85 [0.78, 0.91] |
| Endocrine/Metab | Hyposmolality and/or hyponatremia | Sleep duration | 0.84 [0.79, 0.90] | 0.84 [0.79, 0.90] |
| Endocrine/Metab | Iron deficiency | Sleep duration | 0.90 [0.85, 0.94] | 0.90 [0.85, 0.94] |
| Endocrine/Metab | Protein-calorie malnutrition | Sleep duration | 0.53 [0.41, 0.69] | 0.53 [0.41, 0.69] |
| Endocrine/Metab | Type 2 diabetes | Sleep duration | 0.90 [0.86, 0.93] | 0.89 [0.86, 0.93] |
| Endocrine/Metab | Vitamin D deficiency | Sleep duration | 0.82 [0.75, 0.89] | 0.82 [0.75, 0.89] |
| Gastrointestinal | Constipation | Sleep duration | 0.88 [0.84, 0.92] | 0.88 [0.84, 0.92] |
| Gastrointestinal | Inguinal hernia | Sleep duration | 0.89 [0.84, 0.94] | 0.89 [0.84, 0.94] |
| Genitourinary | Acute kidney failure | Sleep duration | 0.83 [0.79, 0.87] | 0.83 [0.79, 0.87] |
| Genitourinary | Retention of urine | Sleep duration | 0.85 [0.81, 0.90] | 0.85 [0.81, 0.90] |
| Genitourinary | Urinary incontinence and enuresis | Sleep duration | 0.77 [0.71, 0.83] | 0.77 [0.71, 0.83] |
| Genitourinary | Urinary tract infection [UTI] | Sleep duration | 0.82 [0.78, 0.87] | 0.82 [0.78, 0.87] |
| Infections | Bacterial infections | Sleep duration | 0.86 [0.83, 0.90] | 0.86 [0.83, 0.90] |
| Infections | Escherichia coli | Sleep duration | 0.80 [0.73, 0.87] | 0.80 [0.73, 0.87] |
| Mental | Current tobacco use and nicotine dependence | Sleep duration | 0.85 [0.80, 0.91] | 0.85 [0.80, 0.91] |
| Mental | Delirium | Sleep duration | 0.71 [0.66, 0.77] | 0.71 [0.66, 0.77] |
| Mental | Neurocognitive disorder* | Sleep duration | 0.62 [0.51, 0.75] | 0.62 [0.51, 0.75] |
| Mental | Patient's noncompliance with medical treatment and regimen | Sleep duration | 0.64 [0.53, 0.76] | 0.64 [0.53, 0.77] |
| Mental | Psychoactive substance dependance | Sleep duration | 0.68 [0.57, 0.80] | 0.68 [0.58, 0.80] |
| Muscloskeletal | Acquired deformities of limbs | Sleep duration | 0.48 [0.34, 0.68] | 0.48 [0.34, 0.68] |
| Muscloskeletal | Osteoarthritis | Sleep duration | 0.92 [0.89, 0.94] | 0.92 [0.89, 0.94] |
| Muscloskeletal | Osteoporosis | Sleep duration | 0.86 [0.82, 0.91] | 0.86 [0.82, 0.91] |
| Neurological | Abnormality of gait and mobility | Sleep duration | 0.76 [0.72, 0.81] | 0.76 [0.71, 0.81] |
| Neurological | Altered mental status, unspecified | Sleep duration | 0.80 [0.73, 0.87] | 0.79 [0.73, 0.86] |
| Neurological | Dementias | Sleep duration | 0.66 [0.59, 0.73] | 0.66 [0.59, 0.73] |
| Neurological | Parkinson's disease (Primary) | Sleep duration | 0.66 [0.60, 0.74] | 0.66 [0.60, 0.74] |
| Neurological | Parkinsonism | Sleep duration | 0.47 [0.33, 0.67] | 0.47 [0.33, 0.67] |
| Neurological | Repeated falls* | Sleep duration | 0.74 [0.70, 0.79] | 0.75 [0.70, 0.79] |
| Respiratory | Acute lower respiratory infection | Sleep duration | 0.84 [0.80, 0.89] | 0.84 [0.80, 0.89] |
| Respiratory | Chronic obstructive pulmonary disease [COPD] | Sleep duration | 0.84 [0.79, 0.88] | 0.84 [0.79, 0.88] |
| Respiratory | Lobar pneumonia* | Sleep duration | 0.84 [0.80, 0.89] | 0.84 [0.80, 0.89] |
| Symptoms | Allergy to antibiotic agent (excluding penicillin) | Sleep duration | 0.87 [0.82, 0.93] | 0.87 [0.82, 0.93] |
| Symptoms | Edema | Sleep duration | 0.82 [0.76, 0.89] | 0.82 [0.76, 0.89] |
| Symptoms | General symptoms and other findings | Sleep duration | 0.86 [0.82, 0.90] | 0.86 [0.82, 0.90] |
| Symptoms | Malaise and fatigue | Sleep duration | 0.85 [0.79, 0.92] | 0.85 [0.79, 0.92] |
| Genitourinary | Benign prostatic hyperplasia | WASO | 1.10 [1.06, 1.15] | 1.10 [1.06, 1.15] |
| Mental | Alcohol abuse and dependence | WASO | 1.23 [1.14, 1.33] | 1.22 [1.13, 1.32] |
| Mental | Psychoactive substance abuse | WASO | 1.21 [1.11, 1.32] | 1.20 [1.11, 1.31] |
| Mental | Psychoactive substance dependance | WASO | 1.33 [1.16, 1.52] | 1.33 [1.16, 1.52] |
| Muscloskeletal | Osteoarthritis | WASO | 1.05 [1.03, 1.08] | 1.05 [1.03, 1.08] |
| Neurological | Progressive supranuclear ophthalmoplegia [Steele-Richardson-Olszewski]* | WASO | 1.79 [1.36, 2.37] | 1.80 [1.36, 2.38] |
| Gastrointestinal | Abdominal pain | Sleep irregularity | 1.13 [1.07, 1.19] | 1.13 [1.07, 1.19] |
| Mental | Anxiety and anxiety disorders | Sleep irregularity | 1.23 [1.15, 1.31] | 1.23 [1.16, 1.31] |
| Mental | Major depressive disorder | Sleep irregularity | 1.26 [1.18, 1.33] | 1.26 [1.18, 1.34] |
