# Supplementary material for: Accelerometer-derived real-world sleep stages and risk of incident diseases: A UK Biobank cohort study and phenome-wide association analysis
Source: PLoS Med. 2026 Sep 17;23(9):e1005213. doi: 10.1371/journal.pmed.1005213 (PMC13585268; doi:10.1371/journal.pmed.1005213)
Supplement: Supplementary file 17 [file pmed.1005213.s019.docx]

**S12 Table. Associations between sleep irregularity and disease phenotypes with adjustment for total sleep duration**

| **Category** | **Disease Phenotype** | **Sleep Pattern** | **HR [95% CI]** | **HR [95% CI] without adjustment** |
| --- | --- | --- | --- | --- |
| Gastrointestinal | Abdominal pain | Sleep irregularity | 1.15 [1.09 to 1.22] | 1.23 [1.07 to 1.19] |
| Mental | Anxiety and anxiety disorders | Sleep irregularity | 1.25 [1.16 to 1.34] | 1.23 [1.16 to 1.31] |
| Mental | Major depressive disorder | Sleep irregularity | 1.34 [1.25 to 1.43] | 1.26 [1.18 to 1.34] |
