# Supplementary material for: Accelerometer-derived real-world sleep stages and risk of incident diseases: A UK Biobank cohort study and phenome-wide association analysis
Source: PLoS Med. 2026 Sep 17;23(9):e1005213. doi: 10.1371/journal.pmed.1005213 (PMC13585268; doi:10.1371/journal.pmed.1005213)
Supplement: Supplementary file 18 [file pmed.1005213.s020.docx]

**S13 Table. Correlations among repeated measures of sleep patterns**

| **Variable** | **T1 Mean (SD)** | **T2 Mean (SD)** | **Correlation** | ***P*-value** |
| --- | --- | --- | --- | --- |
| REM ^a^ sleep | 89.72 (36.59) | 75.17 (36.82) | 0.62 | <0.001 |
| Deep sleep | 104.86 (34.82) | 100.81 (39.40) | 0.60 | <0.001 |
| Light sleep | 201.04 (48.67) | 185.32 (62.91) | 0.48 | <0.001 |
| WASO ^b^ | 71.84 (37.07) | 64.21 (36.47) | 0.51 | <0.001 |
| Sleep duration | 395.63 (58.89) | 361.29 (95.23) | 0.36 | <0.001 |
| Sleep irregularity | 82.43 (48.04) | 97.31 (60.94) | 0.13 | <0.001 |
